# DJ4 Targets the Rho-Associated Protein Kinase Pathway and Attenuates Disease Progression in Preclinical Murine Models of Acute Myeloid Leukemia

**DOI:** 10.3390/cancers13194889

**Published:** 2021-09-29

**Authors:** Upendarrao Golla, Melanie A. Ehudin, Charyguly Annageldiyev, Zheng Zeng, Diwakar Bastihalli Tukaramrao, Anna Tarren, Abhijit A. Date, Irina Elcheva, Arthur Berg, Shantu Amin, Thomas P. Loughran, Mark Kester, Dhimant Desai, Sinisa Dovat, David Claxton, Arati Sharma

**Affiliations:** 1 Department of Medicine, Division of Hematology and Oncology, Pennsylvania State University College of Medicine, Hershey, PA 17033, USA; ugolla@pennstatehealth.psu.edu (U.G.); cannageldiyev@pennstatehealth.psu.edu (C.A.); atarren@pennstatehealth.psu.edu (A.T.); dclaxton@pennstatehealth.psu.edu (D.C.); 2 Penn State Cancer Institute, Pennsylvania State University College of Medicine, Hershey, PA 17033, USA; asb17@psu.edu (A.B.); samin@pennstatehealth.psu.edu (S.A.); ddesai@pennstatehealth.psu.edu (D.D.); 3 Division of Hematology and Oncology, Department of Pediatrics, Pennsylvania State University College of Medicine, Hershey, PA 17033, USA; mehudin@pennstatehealth.psu.edu (M.A.E.); dbastihallitukaramra@pennstatehealth.psu.edu (D.B.T.); ielcheva@pennstatehealth.psu.edu (I.E.); 4 Department of Pharmacology, Pennsylvania State University College of Medicine, Hershey, PA 17033, USA; beigangmm@hotmail.com (Z.Z.); mk5vq@virginia.edu (M.K.); 5 The Daniel K. Inouye College of Pharmacy, University of Hawaii, Hilo, HI 96720, USA; dateabhi@hawaii.edu; 6 Department of Medicine, Division of Hematology and Oncology, University of Virginia School of Medicine, Charlottesville, VA 22903, USA; tl7cs@virginia.edu; 7 Department of Medicine, Division of Hematology and Oncology, University of Virginia Cancer Center, Charlottesville, VA 22903, USA

**Keywords:** Rho-associated protein kinase, acute myeloid leukemia, DJ4, cell line-derived xenograft, primary human AML cells, preclinical AML murine model

## Abstract

**Simple Summary:**

Acute myeloid leukemia (AML) is a rapidly progressing cancer of the blood and bone marrow with high relapse rates. Standard AML treatment has evolved to yield more frequent remission for patients, with little effect on the disease’s low five-year survival rate. Patients exhibit a wide variation of molecular alterations, driving efforts to profile patients based on these genetic mutations. Previously, our group developed a novel Rho-associated protein kinase (ROCK) inhibitor, DJ4, and biochemical analysis demonstrated its potency in human cancer cell lines. This work targets the overactive ROCKs, which will help patients that experience abnormalities with ROCK-related processes that have been correlated to various cancers. We provide evidence to support the therapeutic efficacy of DJ4 and indicate its promise to improve AML therapy. Our results indicate that inhibiting ROCK makes AML cells susceptible to cell death and, in leukemia mouse models, reduces disease progression and enhances survival.

**Abstract:**

The poor prognosis of acute myeloid leukemia (AML) and the highly heterogenous nature of the disease motivates targeted gene therapeutic investigations. Rho-associated protein kinases (ROCKs) are crucial for various actin cytoskeletal changes, which have established malignant consequences in various cancers, yet are still not being successfully utilized clinically towards cancer treatment. This work establishes the therapeutic activity of ROCK inhibitor (5*Z*)-2–5-(1H-pyrrolo[2,3-*b*]pyridine-3-ylmethylene)-1,3-thiazol-4(5H)-one (DJ4) in both in vitro and in vivo preclinical models of AML to highlight the potential of this class of inhibitors. Herein, DJ4 induced cytotoxic and proapoptotic effects in a dose-dependent manner in human AML cell lines (IC_50_: 0.05–1.68 μM) and primary patient cells (IC_50_: 0.264–13.43 μM); however, normal hematopoietic cells were largely spared. ROCK inhibition by DJ4 disrupts the phosphorylation of downstream targets, myosin light chain (MLC2) and myosin-binding subunit of MLC phosphatase (MYPT), yielding a potent yet selective treatment response at micromolar concentrations, from 0.02 to 1 μM. Murine models injected with luciferase-expressing leukemia cell lines subcutaneously or intravenously and treated with DJ4 exhibited an increase in overall survival and reduction in disease progression relative to the vehicle-treated control mice. Overall, DJ4 is a promising candidate to utilize in future investigations to advance the current AML therapy.

## 1. Introduction

Investigations into the malignant hematopoietic transformation (e.g., myeloid cell abnormal proliferation and abnormal differentiation) of acute myeloid leukemia (AML) are of upmost importance given this disease’s low overall survival, especially among elderly patients [1,2]. The high death rate is often a result of mutations that form a difficult -to-treat heterogenous tumor microenvironment [1,3]. With the current therapy, AML patients frequently enter remission, but nearly always relapse and tend to develop resistance to most existing standard-of-care treatments (e.g., induction cytotoxic chemotherapy or venetoclax-based therapy) [1,4,5,6]. Current targeted therapies are directed towards gene mutations found in AML, such as FMS-like tyrosine kinase 3 (FLT3) [7,8,9], KIT [10,11], BCR-ABL [12], *TP53* [13,14], isocitrate dehydrogenase (IDH1 and IDH2) [1,15,16], or mixed lineage leukemia (MLL) [17,18,19]. Further, targeted therapy is aimed at inhibiting signaling pathways involving aurora kinases (AURKs) [20,21,22], polo-like kinases (PLKs) [23,24], hedgehog (Hh) [25,26,27] or Rho kinases (ROCKs) [28,29,30,31], or even surface antigens such as CD33 [32] or CD47 [33,34] that tend to be present on myeloid cells. Efforts are ongoing to develop checkpoint inhibitor immunotherapies against programmed death 1 (PD-1) or programmed death ligand 1 (PD-L1) proteins to help stimulate the patient’s immune system to fight cancer cells [35]. Despite these innovations, there are many common obstacles to improving the status quo drug regimen and the current prognosis of AML patients. These include establishing whether a therapy is potent enough to inhibit its target to an effective level, optimizing the dosing for effective treatment response while avoiding chemoresistance, determining which drug and/or immunotherapy combination is most efficacious, or evaluating when to administer a certain treatment (better as induction or consolidation therapy). Determining a targeted therapy regimen unique to the patient can be complicated by the time needed to examine the patient’s leukemic mutation profile and avoid toxicity to normal hematopoietic and nonhematopoietic tissues in the balance of their disease progression [1].

Interactions between Rho GTPases and the downstream effector, Rho-associated coiled-coil-containing protein kinases (ROCK1 and ROCK2) allow for the phosphorylation of various substrates including the myosin light chain (MLC2) and the myosin-binding subunit of MLC phosphatase (MYPT) which promote actin cytoskeletal changes [36,37]. The Rho-ROCK pathway regulates essential biological processes relating to cell morphology, shape, contraction, migration, adhesion, motility, proliferation, differentiation, cell junction integrity, cell cycle control, and apoptosis [37,38,39,40]. This signaling network has established oncogenic roles, with consequences toward tumor development and progression, metastasis, motility, invasion, apoptosis and survival, tumor microenvironment, and angiogenesis [39,41,42,43,44]. Recent advances have also explored utilizing short interfering RNA (siRNA)- or short hairpin RNA (shRNA)-based gene silencing methods to further understand the role of ROCK in various diseases [39,42,45]. However, due to its broad functionality and the high overall identity between the ROCK1 and ROCK2 isoforms, studies are still needed to fully understand its advantageous or disadvantageous roles in these signaling pathways, complicating selective ROCK inhibition drug development. Further, current ROCK inhibitors such as Fasudil or Y27632 (not used as the current standard of care cancer treatment) tend to have multikinase activity and therefore may have potential off-target effects [39,41,43,46]. Thus, recent advances are aimed at not only designing more selective inhibitors, but also at developing appropriate chemotherapeutics to use in combination to support clinical utility of these ROCK inhibitors towards various cancers [39,41,43,46,47]. There is also a need for further preclinical studies of ROCK inhibitor anticancer therapy to determine which cancers (e.g., tumor cell type and microenvironment) or which types of patients this treatment would be most efficacious for and to establish pharmacodynamic or characteristic biomarker endpoints of treatment [39,41].

Targeting several areas of a signaling cascade or multiple kinases with similar functions can provide a more efficacious treatment, one that is less susceptible to chemoresistance; for instance, ROCK and MRCK combined inhibition has been previously reported to be more potent in inhibiting actomyosin-regulated functions [41,48,49]. Additionally, lung cancer cell line A549 and breast cancer cell line MDA-MB-231 have been shown to be sensitive to DJ4 [50]. Western blotting, kinase functional/cellular activity, and computational assays indicated DJ4 selectively acts as an ATP competitive inhibitor of the kinases ROCK1/2 and MRCKα/β, which are responsible for the processes needed for cancer cell migration/invasion [50]. These findings indicate the promising therapeutic potential of DJ4 in vitro in human lung and breast cancer cell lines [50]; however, in vitro and vivo efficacy in AML has not been pursued.

Herein, continuing efforts to thoroughly examine the therapeutic efficacy of (5*Z*)-2–5-(1H-pyrrolo[2,3-*b*]pyridine-3-ylmethylene)-1,3-thiazol-4(5H)-one (DJ4), a ROCK and myotonic dystrophy kinase-related Cdc42-binding kinase (MRCK) inhibitor [50], towards acute myeloid leukemia are presented. We describe cell proliferation and colony formation assays followed by preclinical murine therapeutic studies to gauge the potency of DJ4 towards various AML cell lines. AML patient-derived samples were also studied to determine whether the treatment prevents tumor formation or progression towards a heterogenous lineage which is characteristic of this disease. Further, apoptotic assays, Western blotting, and flow cytometry analysis were performed to gain molecular insight into the downstream processes and therapeutic benefit achieved by DJ4. This work highlights the potential of targeting the Rho-ROCK pathway to improve the prognosis of AML. The experiments herein present an analysis to establish a ROCK inhibitor, thereby laying the fundamental groundwork needed for the future development of chemotherapeutics that are both less toxic and more effective.

## 2. Materials and Methods

### 2.1. Cells and Cell Culture

The human AML-derived cell lines HL-60 (CCL-240), MV4-11 (CRL-9591) were obtained from the American Type Culture Collection (ATCC), Manassas, VA, USA. All the other cell lines such as OCI-AML2, OCI-AML3, MOLM-13, and U937 were provided to us as mentioned in the acknowledgments. Cell lines utilized in this study were authenticated by short tandem repeat (STR) profiling. Bone marrow aspirates or peripheral blood samples were acquired from AML patients, and cord blood (CB) samples were obtained from freshly delivered placentas of healthy donors after informed consent using protocols approved by the Institutional Review Board of the Penn State College of Medicine. Mononuclear cells (MNC) were isolated by means of density gradient separation (Ficol-Paque, GE Healthcare Life Sciences, Pittsburgh, PA, USA). Cell lines and primary cells were cultured as previously described [51,52]. DJ4 was synthesized previously at the Organic Synthesis Core of the Penn State College of Medicine, and its high purity (>99%) was quantified via high-performance liquid chromatography and nuclear magnetic spectroscopy [50]. DJ4 was dissolved in dimethyl sulfoxide (DMSO) and filtered through a 0.22-micron filter prior to cell or animal treatment. The percentage of DMSO was < 0.01% for the in vitro assays.

### 2.2. Cell Viability Assay

The cells were seeded at a constant density in a 96-well plate and treated with increasing concentrations of DJ4 (0.001–20 μM) for 24 h. Each concentration was tested in triplicate within the plate. The relative viability and IC_50_ were measured using the MTS [3-(4,5-dimethylthiazol-2-yl)-5-(3-carboxymethoxyphenyl)-2-(4-sulfophenyl)-2H-tetrazlium, inner salt] assay (CellTiter 96 Aqueous One Solution Cell Proliferation Assay, Promega, Madison, WI, USA) according to the manufacturer’s protocol and the data were analyzed using the GraphPad Prism 6.0 software (GraphPad Software, San Diego, CA, USA). Baseline absorbance at 490 nm was subtracted from the data and normalized to the controls. Cytotoxicity experiments were conducted in three independent trials to ensure reproducibility.

### 2.3. Apoptotic and Cell Cycle Assays

The cells were plated at a constant seeding density and treated with increasing concentrations of DJ4 (0–20 μM) for 24 h. The percentage of apoptosis in AML cells was detected using the combination of the AnnexinV-PE (BD Biosciences, Franklin Lakes, NJ, USA) and 7-AAD (BioLegend, San Diego, CA, USA) dyes. Additionally, the cells were fixed with ethanol after 24 h treatment with DJ4 for cell cycle analysis using the FxCycle™ PI/RNase Staining Solution (Thermo Fisher Scientific, Waltham, MA, USA). The data for apoptosis and cell cycle analysis were acquired using a BD Accuri™ C6 Plus instrument. The relative apoptosis of the primary AML patient cells was determined by resuspending and incubating the cells using the Muse Annexin V & Dead Cell Kit (MCH100105, Millipore, Burlington, MA, USA) according to the manufacturer’s protocol. The values were obtained using a benchtop flow cytometer, Muse Cell Analyzer (Millipore Sigma).

### 2.4. Colony-Forming Assay

The cells were cultured at a constant optimal seeding density in Human Methylcellulose Base Media (R&D Systems, Minneapolis, MN, USA) in a 12-well plate to yield colony outgrowth of 20–100 colonies per well as described previously [51,52]. To test the clonogenic potential, the cells were cultured in the presence of increasing concentrations of DJ4 (0–10 μM) or the DMSO vehicle in a methylcellulose medium for 7–14 days. Blast colonies (>20 cells/colony) were counted under a light microscope and imaged with an Olympus CKX31 inverted microscope (Olympus Corporation, Center Valley, PA, USA) using a 4× objective.

### 2.5. Western Blot Analysis

The AML cell line (MV4-11 and OCI-AML3) cells were treated with increasing concentrations of DJ4 (0–1 μM) or DMSO and harvested at 24 h. Whole cell lysates were then collected in a RIPA buffer (Sigma) containing phosphatase and protease inhibitor cocktails (Sigma). Protein quantification was performed using a bicinchoninic acid (BCA) assay kit (Pierce, Thermo Fisher Scientific, Waltham, MA, USA). Denatured protein samples run on a NuPAGE 4–12% Bis-Tris gel (Life Technologies, Carlsbad, CA, USA) were subsequently probed with various primary antibodies as previously described [51,52]. Bands were detected using the Bio-Rad ChemiDoc MP imaging system (Bio-Rad Laboratories, Hercules, CA, USA) and quantified via the ImageJ software [53]. The following antibodies were obtained: ROCK1 (611136; BD Biosciences), ROCK2 (610623; BD Biosciences), MYPT1 (07-672-I; Millipore Sigma), Phospho-MYPT1 (Thr696) (5163S; Cell Signaling Technology, Danvers, MA, USA), MLC2 (3672S; Cell Signaling Technology), Phospho-MLC2 (PA5-17726; Thermo Scientific), and GAPDH (sc-32233; Santa Cruz Biotechnology, Dallas, TX, USA); the goat anti-rabbit IgG–horseradish peroxidase (HRP) conjugates and the horse anti-mouse IgG-HRP conjugates were purchased from Cell Signaling Technology. All the whole western blot figures can be found in the Figure S9.

### 2.6. Preclinical Murine Studies

Toxicity, dosing, and route of DJ4 were assessed by treating B6(Cg)-*Tyr^c−2J^*/J (Albino B6) mice (The Jackson Laboratory, Bar Harbor, ME, USA) with DJ4 and monitoring body weight, survival, and overt signs of illness. The mice were administered the vehicle DMSO (*n* = 5) or a solution of DJ4 in DMSO (10 mg/kg, *n* = 5) intraperitoneally (I.P.) for 2.5 weeks. The animals were then euthanized, and blood was collected via cardiac puncture for complete blood count (CBC) with differential and chemistries. The CBC with differential measured the levels of white blood cells (WBCs), red blood cells (RBCs), hemoglobin (Hgb), hematocrit (HCT), mean corpuscular volume (MCV), mean corpuscular hemoglobin (MCH), mean corpuscular hemoglobin concentration (MCHC), and platelets. The metabolic panel consisted of the following tests: glucose (GLU), creatinine (CREA), blood urea nitrogen (BUN), phosphorus (PHOS), calcium (CA), total protein (TP), albumin (ALB), alanine aminotransferase (ALT), alkaline phosphatase (ALKP), total bilirubin (TBIL), total cholesterol (CHOL), and amylase (AMYL). Cross-sections of the spleen, liver, lung, and kidney were formalin-fixed and paraffin-embedded to assess tissue morphology with hematoxylin and eosin (H&E) staining. Images of the tissue morphology and the stained section were captured using Nikon Eclipse Ts2R (Nikon, Melville, NY, USA).

Pharmacokinetic data of DJ4 were established by intraperitoneally treating 21 albino B6 mice with DJ4 (10 mg/kg) and collecting blood via cardiac puncture from three mice at each of the following time points: 0.25, 0.5, 1, 3, 6, 12, and 24 h. Blood was processed to isolate the serum and then submitted for mass spectrometry analysis to quantify the circulating concentration of DJ4 in the blood over time.

Cell line-derived xenograft (CDX) models were established with OCI-AML3–yellow fluorescent protein (YFP)–luciferase (Luc) and MV4-11-Luc2–enhanced green fluorescent protein (EGFP). The luciferase-expressing leukemia cell lines were subcutaneously (S.C., 2–2.5 × 10^6^ cells) or intravenously (I.V., 2–2.5 × 10^6^ cells) administered to 8–12-week-old NOD.Cg-*Rag1^tm1Mom^ Il2rg^tm1Wjl^* Tg(CMV-IL3,CSF2,KITLG)1Eav/J (NRG-S) mice (The Jackson Laboratory, Bar Harbor, ME, USA) and the disease progression was monitored with bioluminescence imaging (BLI) as previously established [51,52]. The mice were randomized based on the BLI signal, segregated into either control or treatment groups, and intraperitoneally treated with DMSO or DJ4 (10 mg/kg) for 3 weeks. The in vivo studies initially included extra mice, with outliers in engraftment being removed, to ensure no variability in the leukemia burden among the mice within the control and DJ4 groups prior to randomization. Treatment was performed in a continuous cycle administering DJ4 once a day for five days followed by a two-day break. The murine studies were repeated at least twice. Whole-body leukemic burden was quantified using the Living Image software (Perkin Elmer). Overall survival was monitored, and Kaplan–Meier analysis was carried out. Over the course of the study (subcutaneous model studies), the tumor volume (mm^3^) was measured with calipers, and towards the conclusion of the study, the tumors were isolated and weighed (g).

The luciferase-labeled cell lines were also treated with DMSO or their respective IC_50_ dose of DJ4 for 24 h and administered intravenously to the NRG-S mice. The mice injected with the pretreated cells were then monitored without further treatment, and similar imaging and survival analysis was performed. Bone marrow and spleen tissues were additionally harvested from the DMSO and DJ4 pretreated groups and analyzed for engraftment by flow cytometry. The tissues harvested from the murine studies were evaluated by staining for GFP expression, APC-Cy7-labeled anti-human CD45 (hCD45, BioLegend), mouse CD45-BV650 (mCD45, BD Bioscience, Billerica, MA, USA), and dead cell exclusion dye, 7AAD (BioLegend). The data were collected by means of flow cytometry using a BD LSR II flow cytometer and analyzed via the FlowJo software [54].

All the animal experiments were conducted at the Penn State University College of Medicine under protocols approved by the Institutional Animal Care and Use Committee at Penn State, Hershey, PA, USA (IACUC # PROTO201246746).

### 2.7. Statistical Analysis

Statistical analysis was performed by means of the *t*-test, Gehan–Breslow–Wilcoxon test, or two-way analysis of variance (ANOVA) utilizing the GraphPad Prism 6.0 software (GraphPad Software, San Diego, CA, USA). All the findings reported herein were repeated in at least two independent experiments and are the means ± standard error of the mean (SEM) or standard deviation (SD), wherein *p* < 0.05 (95% CI) is considered statistically significant; *p*-values or asterisks denote the data that were statistically significant.

## 3. Results

### 3.1. DJ4 Exerts Cytotoxic Activity in AML Cell Lines and Primary Cells

**Figure 1:**
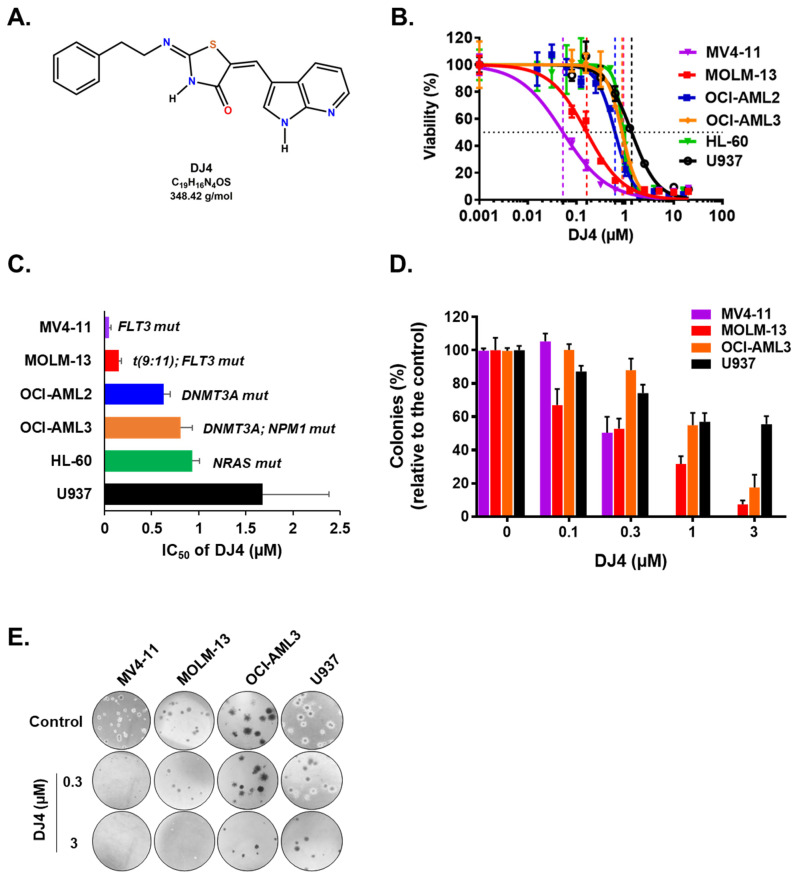
Human AML cell lines are sensitive to DJ4 treatment. (**A**) Chemical structure of DJ4. (**B**) Cytotoxic drug response measured using the MTS assay upon DJ4 treatment for 24 h on various human AML cell lines. (**C**) Corresponding IC_50_ (µM) values from the MTS assay indicative of the sensitivity of cells with various AML-specific mutations to DJ4. (**D**) Colony-inhibiting capability of DJ4 on AML cell lines. (**E**) Microscopic images (4×) of the DJ4-mediated effect on clonogenicity of AML cell lines in a colony growth medium. The data are the means ± standard deviation (SD).

ROCK1 overexpression has been linked to AML cell lines and overall survival of AML patients, suggesting that ROCK inhibition may mediate leukemic cell death and improve conventional AML therapeutics (Figures S1 and S2). To determine the cytotoxic effect of DJ4 (Figure 1A) on AML cells in vitro, several human leukemic cell lines (MOLM-13, MV4-11, OCI-AML2, OCI-AML3, HL-60, and U937) and AML patient samples were chosen. The cell proliferation and relative viability were measured after treatment with DJ4 (0.001–20 μM) for 24 h. For all the AML cell lines, as the concentration of DJ4 was increased, the relative viability decreased in a dose-dependent manner (Figure 1B). The half-inhibitory concentration (IC_50_) values for the cell lines were between 0.05 and 1.68 μM (Figure 1C and Table S1). MV4-11 was the most sensitive AML cell line to drug treatment (IC_50_ = 0.05 ± 0.02 μM), followed by MOLM-13 (IC_50_ = 0.15 ± 0.03 μM), OCI-AML2 (IC_50_ = 0.63 ± 0.07 μM), OCI-AML3 (IC_50_ = 0.81 ± 0.12 μM), and HL-60 (IC_50_ = 0.93 ± 0.05 μM) (Figure 1B and Table S1). The cell line with no specific AML molecular signature, U937, was the least responsive (IC_50_ = 1.68 ± 0.70 μM) to DJ4 treatment.

**Figure 2:**
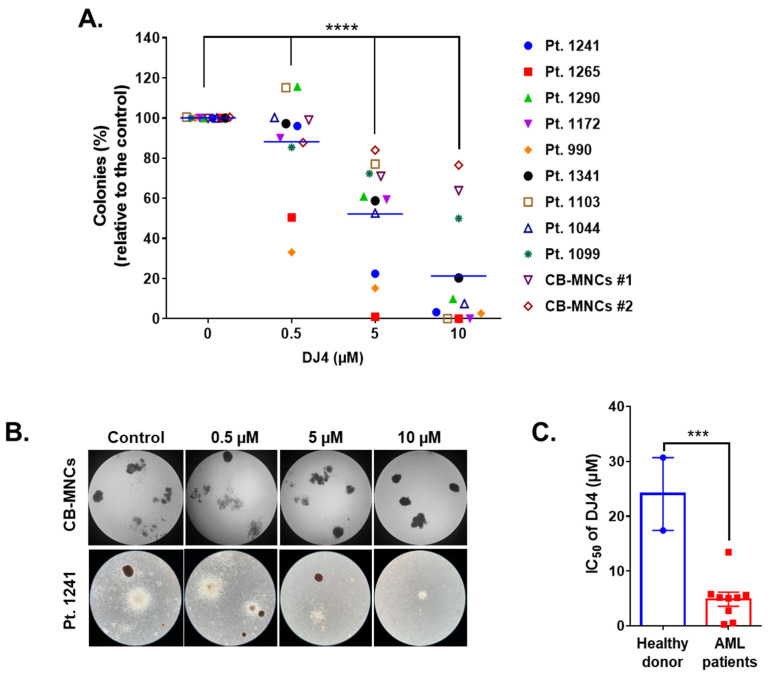
DJ4 reduces clonogenicity in AML patient samples while sparing healthy donor cells. (**A**) Clonogenicity of the primary AML samples relative to the cord blood mononuclear cells (CB-MNCs) in the presence of DJ4. The data were analyzed via two-way ANOVA (Dunnett’s multiple comparisons test) to compare the grand mean colonies, shown with a blue line, of DJ4 treatment versus the control colonies, wherein **** *p* < 0.0001 was considered significant. (**B**) Representative microscopic images (4×) illustrating the number of colonies with increasing DJ4 concentrations in AML primary patient sample 1241 versus the control CB-MNCs. (**C**) Extrapolation of the IC_50_ (µM) values from the colony-forming assay in (**A**). The results were assessed by means of the unpaired *t*-test and *** *p* < 0.001 was considered significant. The data are the means ± SEM.

DJ4 treatment inhibited the colony-forming ability of the AML cell lines (Figure 1D,E) and the primary patient cells (Figure 2) in the micromolar range and a dose-dependent manner. Drug treatment was able to inhibit the number of colonies, particularly of MV4-11 and MOLM-13, wherein approximately half the number of colonies were observed relative to the untreated cells with 0.3 μM DJ4 treatment (Figure 1D,E). A reduction in the colony-forming ability of U937 by ~40–50% required 1–3 μM DJ4 treatment. The AML primary cells had a marked decrease in the colony-forming ability with increasing DJ4 treatment (Figure 2). The IC_50_ values of DJ4 for the cord blood mononuclear cells (CB-MNCs) and the AML primary cells were extrapolated from the colony-forming assay and found to range from 0.26 to 25 μM (Figure 2C and Table 1). In the presence of 0.5 μM DJ4 treatment, AML patient samples 1265 (IC_50_ = 0.50 μM) and 990 (IC_50_ = 0.26 μM) exhibited a considerable reduction of ~50% in the number of colonies relative to the untreated cells (Figure 2A). The other AML patient samples such as 1241 (IC_50_ = 2.77 μM), 1172 (IC_50_ = 5.06 μM), 1103 (IC_50_ = 5.17 μM), 1044 (IC_50_ = 5.14 μM), 1290 (IC_50_ = 5.62 μM), and 1341 (IC_50_ = 5.77 μM) needed higher concentrations of DJ4 (2.5–5 μM) to impact the number of colonies (Figure 2A, Table 1). AML patient sample 1099 (IC_50_ = 13.43 μM) required 10–20 μM DJ4 to affect its colony-forming ability significantly. However, treatment with DJ4 imparted a considerable effect on the AML patient cells relative to the CB-MNCs of the healthy donor cells (IC_50_ = 25 μM) (Figure 2B,C, Table 1). DJ4 was ~5-fold more selective towards the AML primary cells compared to the primary CB-MNCs (Figure 2B,C, Table 1).

### 3.2. DJ4 Induces Apoptosis in AML Cell Lines and Primary Cells

**Figure 3:**
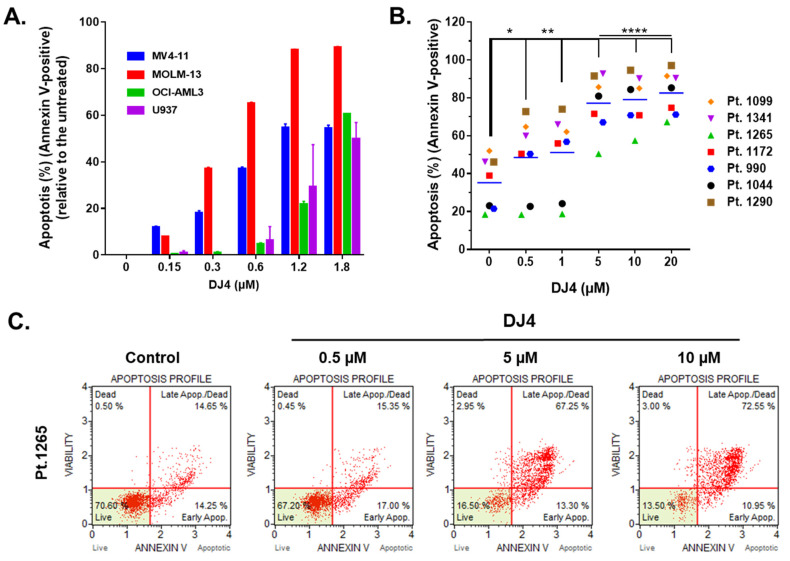
DJ4 treatment exerts a proapoptotic effect in AML cell lines and primary samples. (**A**) Induction of apoptosis was measured as a percentage of Annexin V-positive cells upon treating the human AML cell lines with increasing concentrations of DJ4 for 24 h. The values are reported as the means ± SEM. (**B**) The apoptotic effect in the AML primary patient samples was quantified as the percentage of Annexin V-positive cells after subsequent 24 h DJ4 treatment (see Figure S4 for the normalized plot). The data were analyzed via two-way ANOVA (Dunnett’s multiple comparisons test) to compare the grand mean apoptosis, indicated by the black line, of DJ4 treatment versus the control, wherein * *p* < 0.05, ** *p* < 0.01, **** *p* < 0.0001 were considered significant. (**C**) Representative flow plots of the live, dead, and apoptotic populations (early and late apoptosis) upon treating AML primary cells with DJ4 (0–10 μM). The data are the result of two independent experiments.

Treatment of DJ4 after 24 h on the AML cell lines and the primary AML patient samples induced apoptosis within the micromolar range in a dose-dependent manner (Figure 3 and Figures S3 and S4). MOLM-13 was considerably more sensitive to DJ4 treatment, wherein higher percentage of apoptosis was observed in the presence of 0.3–1.8 μM DJ4, than the other AML cell lines (Figure 3A). Exposure to low concentrations, 0.3 and 0.6 μM, of DJ4 induced about 40% and 70% apoptosis in MOLM-13, respectively. The other AML cell lines required higher DJ4 concentrations to exert the same apoptotic effect. MV4-11 needed 0.6–1.2 μM DJ4 treatment, whereas OCI-AML3 and U937 required exposure to 1.2–1.8 μM DJ4 to lead to ~50% apoptosis (Figure 3A). Cell cycle analysis also demonstrated the impact of DJ4 in the OCI-AML3, MV4-11, and MOML-13 cells to induce cell death via apoptosis by the dose-dependent increase in the sub-G0/G1 cell population with increasing concentrations of the drug (Figure S3). Exposure to DMSO or 0.05, 0.15, 0.3, and 0.6 μM DJ4 of the MV4-11 cells resulted in an increase in the sub-G0/G1 population by 2.90, 3.50, 4.61, 9.55, and 11.9%, respectively (Figure S3A,B). DJ4 enhanced the G0/G1 cell death phase in the MOLM-13 cells by 3.46, 8.63, 9.37, and 11.40% with 0.15, 0.3, 0.6, and 1.2 μM drug treatment, respectively (Figure S3C,D). Treatment with 0.3, 0.6, and 1.2 μM DJ4 in OCI-AML3 resulted in a greater sub-G0/G1 population by 2.03, 3.16, and 8.28%, respectively (Figure S3E,F). A significant change in the apoptotic population was observed in the cell cycle assay with 1.2 μM DJ4 treatment of OCI-AML3, whereas exposure to only 0.3 μM DJ4 was needed to induce a greater apoptotic response in the MV4-11 and MOLM-13 cell lines. This is comparable to the results observed with the MTS and colony-forming assays, wherein MV4-11 and MOLM-13 were more sensitive than OCI-AML3 towards DJ4. The AML cell lines tended to be more sensitive to DJ4 treatment as observed in the cytotoxicity and colony-forming assays requiring less DJ4 to induce 50% apoptosis versus the AML primary cells (Figure 3 and Figure S4). Representative flow cytometry plots of the Annexin V assay with the AML primary cells shown in Figure 3B depict the dose-dependent increase in the apoptotic populations of AML primary sample 1265 as a result of treatment with increasing concentrations of DJ4 (Figure 3C). AML patient sample 990 was the most responsive, with a greater percentage of apoptosis, 30–40%, observed relative to the untreated cells, with low concentrations of DJ4 treatment (0.5–1 μM) than the other AML primary cases (Figure 3B and Figure S4). This is consistent with the colony forming data where patient samples 990 and 1265 were the most sensitive to DJ4 treatment. Treatment with low concentrations of DJ4 (0.5–1 μM) induced 10–20% apoptosis versus the untreated cells in AML primary samples 1172, 1099, 1290, and 1341. Treatment with 5 μM of DJ4 stimulated ~30–50% apoptosis relative to the untreated cells in all the AML primary samples (Figure 3B,C and Figure S4). The apoptotic effect of 10–20 μM versus 5 μM DJ4 treatment was not vastly different among the AML primary samples (Figure 3B,C and Figure S4).

### 3.3. DJ4 Effectively Inhibits the ROCK/MYPT1/MLC2 Pathway

**Figure 4:**
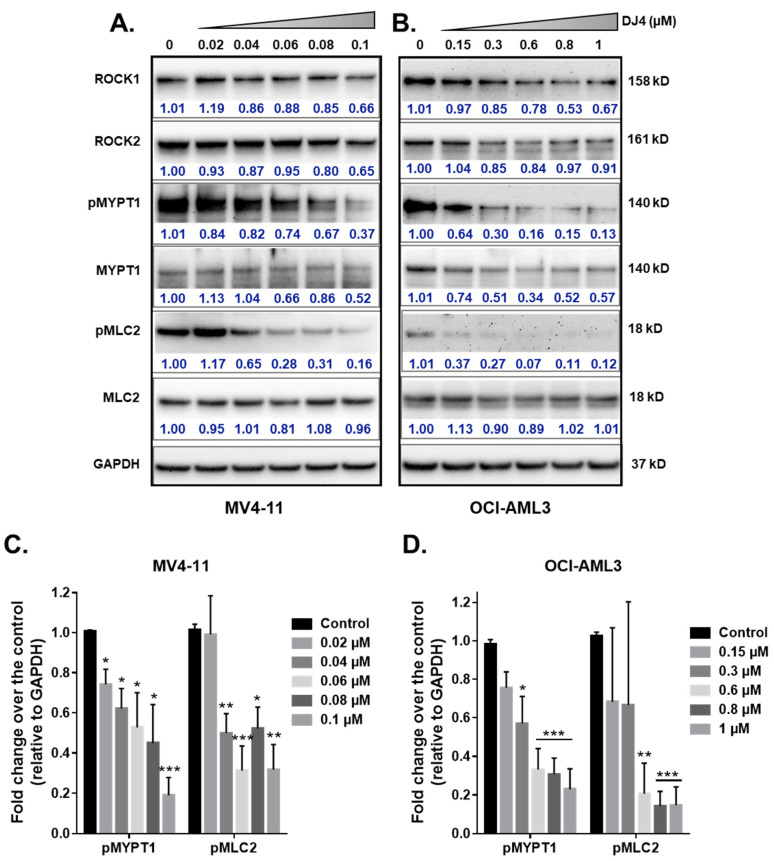
DJ4 effectively inhibits downstream ROCK substrates in human AML cell lines. Immunoblot analysis of the relative activity of MYPT1, MLC2, and expression levels of ROCK1, and ROCK2 upon treating the whole cell lysates of (**A**) MV4-11 and (**B**) OCI-AML3 with DJ4 for 24 h. The phosphorylated levels of MYPT1 and MLC2 relative to the loading control GAPDH in (**C**) MV4-11 and (**D**) OCI-AML3 in the presence of DJ4 were quantified. The data were representative of three independent experiments. The results were assessed by means of the unpaired *t*-test and * *p* < 0.05, ** *p* < 0.01, and *** *p* < 0.005 were considered significant. The data are the means ± SEM (*n* = 3).

The effect of DJ4 treatment on the phosphorylation of ROCK downstream targets, MYPT1 and MLC2, was assessed. The AML cell lines, MV4-11 and OCI-AML3, were treated with DJ4 in DMSO (0–1 μM). The whole cell lysates were prepared after 24 h of drug treatment and analyzed via immunoblot analysis (Figure 4). Upon treating MV4-11 (Figure 4A) and OCI-AML3 (Figure 4B) with increasing concentrations of DJ4, the levels of phosphorylated MYPT1 and MLC2 were reduced in a dose-dependent manner relative to the loading control GAPDH (Figure 4C,D) while retaining the levels of unphosphorylated analogs of these substrates and ROCK1 and ROCK2. Additionally, lower concentrations of DJ4 were needed to significantly reduce the levels of phosphorylated MYPT1 and MLC2 in MV4-11 (0.04–0.06 μM) versus OCI-AML3 (0.6–1 μM). This behavior of increased sensitivity of MV4-11 to DJ4 versus OCI-AML3 is comparable to what was observed with the aforementioned cytotoxicity and apoptotic assays (Figure 1B–E and Figure 3A). This supports the in vitro studies by Kale et al. who demonstrated the potency of DJ4 to selectively inhibit ROCK1/2 and MRCKα/β in various human cancer cell lines. [50]. Similarly, DJ4 inhibited the activity of MYPT1 and reduced the levels of MLC2 [50].

### 3.4. Systemic Administration of DJ4 Is Well-Tolerated by Mice without Adverse Side Effects

**Figure 5:**
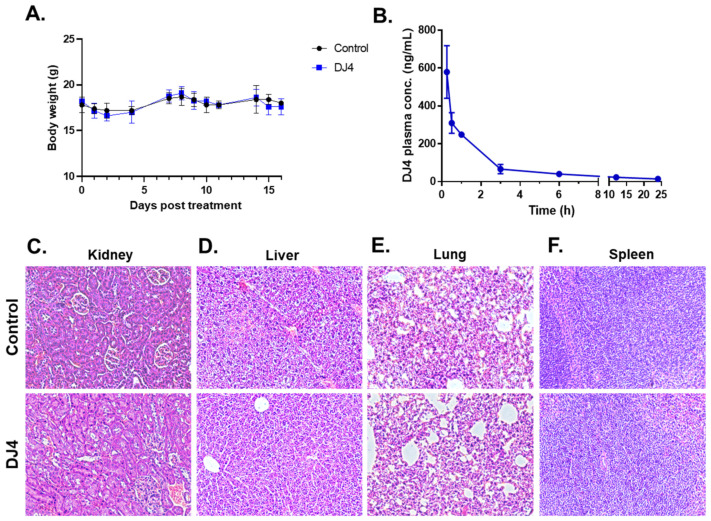
Intraperitoneal administration of 10 mg/kg DJ4 for 2.5 weeks was tolerated in the mice without systemic toxicity. (**A**) The weights (g) of the mice treated with the vehicle versus DJ4 were comparable. The values are represented as the means ± SD (*n* = 5, see Table S2 for the average body weight values). (**B**) Pharmacokinetic analysis to quantify the concentration of DJ4 in the serum of the treated mice at various time points (0.15–24 h). The values are represented as the mean ± SEM (*n* = 3). (**C**–**F**) Cross-sections of kidney (**C**), liver (**D**), lung (**E**), and spleen (**F**) tissues from the mice treated with DMSO (top) and DJ4 (bottom) that were formalin-fixed, paraffin-embedded, and hematoxylin-and eosin-stained to examine the cell or organ morphology. Magnification: 20×.

DJ4 was formulated in DMSO; thus, the maximum tolerated dose (MTD) was conducted via intraperitoneal injection. The MTD was established to be 10 mg/kg. The mice were then treated with the DMSO vehicle (*n* = 5) and 10 mg/kg DJ4 (*n* = 5) I.P. for 2.5 weeks, wherein the drug was administered once a day for 5 days with a 2-day break in a cycle. Drug treatment had a negligible impact on the body weight (less than 10% of the body weight lost), and no signs of illness were observed over time (Figure 5A, Table S2). The control and DJ4 treatment groups had comparable complete blood counts (CBC) with differential values suggesting drug treatment did not cause severe underlying conditions (Table S3). A metabolic panel to analyze renal function (CREA and BUN), liver function (ALKP, ALT, and AST) and common electrolytes in the blood collected from the control and DJ4 treatment groups was also examined (Table S4). The similar values from the chemistry panel between the groups indicate the levels of glucose, fluid, and electrolytes or the function of kidneys, liver, and other organs were not adversely affected by DJ4 (Table S4). Pharmacokinetic analysis indicated that approximately one hour after I.P. administration of DJ4 (10 mg/kg) ~50% of the drug remained in the blood, and after three to six hours, the level of DJ4 in the blood was negligible (Figure 5B). Further, H&E staining of the kidney, liver, lung, and spleen tissues of the mice treated I.P. for 2.5 weeks with DMSO or DJ4 indicated the cell and organ morphology was not affected by drug treatment (Figure 5C–F). Thus, the mice tolerated 10 mg/kg DJ4 via I.P. administration without any overt signs of illness and harmful effects to their system.

### 3.5. DJ4 Reduces Disease Progression and Enhances Survival in AML CDX Murine Models

**Figure 6:**
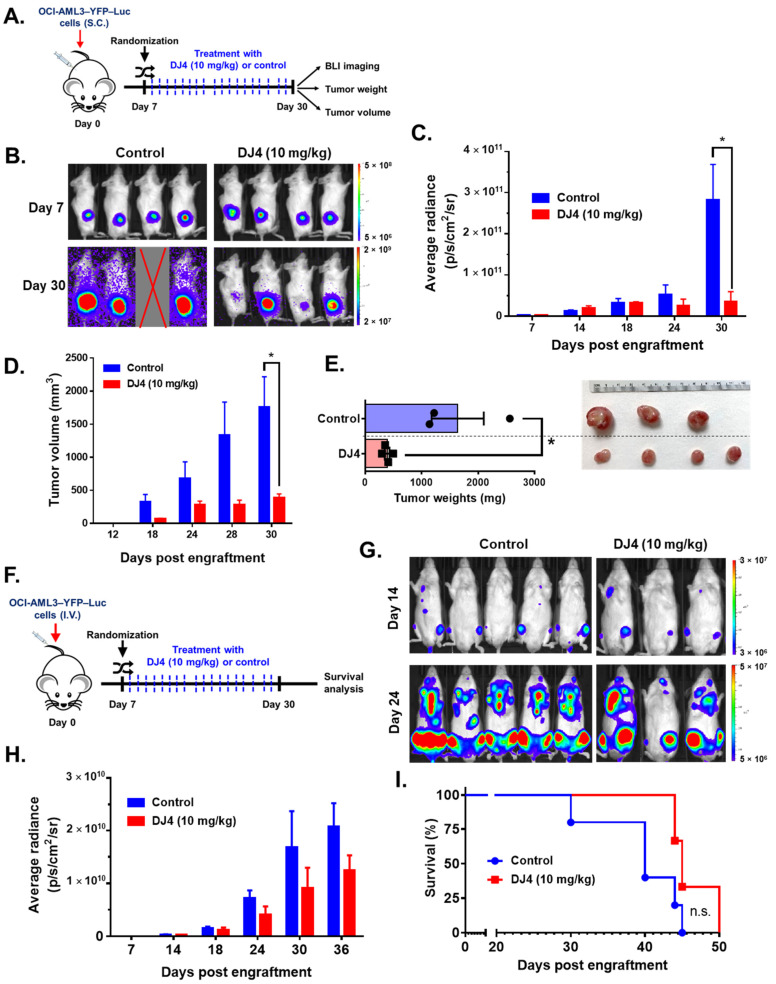
DJ4 administration (10 mg/kg) for three weeks was efficacious in the disseminated and subcutaneous models of OCI-AML3–YFP–Luc-injected mice. (**A**) The labeled OCI-AML3 cells were administered subcutaneously to the immunocompromised NRG-S mice and segregated into treatment groups based on their bioluminescent signal on day seven. The mice were subdivided in a manner to ensure the average signal between both groups was comparable at the start of the study and then treated with either the vehicle or DJ4. The average radiance (p/s/cm^2^/sr), tumor volume (mm^3^), and tumor weights (mg) of the animals were measured over time. The results were assessed by means of the unpaired *t*-test, wherein * *p* < 0.05 was considered statistically significant. (**B**) Images of the subcutaneous tumor of the control and DJ4-treated mice on days 7 and 30. (**C**) Bioluminescent signal of the DJ4-treated mice relative to the control group over time. (**D**) Volumes of the subcutaneous tumors in the mice that were administered DJ4 as compared to the vehicle-treated mice over the course of the study. (**E**) The tumor weights of the DJ4-treated mice and the gross examination of the tumor sizes in the groups. (**F**) The immunocompromised NRG-S mice were intravenously injected with the OCI-AML3–YFP–Luc cells. The mice were randomized into the vehicle- or DJ4-treated group based on their bioluminescent signal on day seven to ensure the average intensity was similar in both groups at the start of the study. The mice were intraperitoneally treated with the vehicle or DJ4 for 3 weeks and the survival advantage of drug treatment was monitored. IVIS imaging of the mice took place every few days. (**G**) Images of the DJ4-treated mice relative to the control group on days 14 and 24. (**H**) Bioluminescent signal measured as the average radiance of the DJ4-treated mice versus the control group as observed over time. The values were examined by means of the *t*-test, and * *p* < 0.05 was considered statistically significant. (**I**) Kaplan–Meier survival analysis of the DJ4 group versus the vehicle-treated group. The results were assessed by means of the Gehan–Breslow–Wilcoxon test. The data are the means ± SEM; n.s. denotes not significant.

Efficacy of intraperitoneal DJ4 administration (10 mg/kg) in tumor-bearing mice was assessed utilizing the AML cell lines relatively sensitive to drug treatment, OCI-AML3 and MV4-11. The cell line-derived xenograft models, OCI-AML3–YFP–Luc and MV4-11–Luc2–EGFP, were established utilizing flow cytometry, wherein the YFP/GFP expression was greater than 95% relative to the unlabeled cells (Figure S5). Further, MTS cell proliferation assay showed no significant differences in DJ4 treatment response between the labeled and unlabeled AML cell lines (Figure S6). The CDX models were then optimized (e.g., cell number) in the immunocompromised NRG-S mice prior to therapeutic studies. The mice subcutaneously injected with OCI-AML3–YFP–Luc were treated with the vehicle DMSO (*n =* 5) or 10 mg/kg DJ4 (*n =* 5) for three weeks (Figure 6A). The bioluminescent signals or average radiance (p/s/cm^2^/sr) of the subcutaneously injected OCI-AML3–YFP–Luc mice were comparable on day seven between the control and treatment groups (Figure 6B,C), though on day thirty, a significant, at least threefold reduction in the average radiance was observed in the DJ4-treated group relative to the control group (Figure 6B,C). After day 12, a significant difference was observed in the tumor volume between the control and treatment groups (Figure 6D). The tumor volume on day 30 was significantly, ~4-fold lower in the DJ4-treated OCI-AML3–YFP–Luc mice versus the control group (Figure 6D), and the tumor weight was significantly reduced (~3-fold) (Figure 6E). On day 30, the average tumor volume and tumor weight of the control group were ~2000 mm^3^ and ~1700 mg in comparison to the smaller values shown for the DJ4-treated group where average tumor volume and weight of ~500 mm^3^ and ~500 mg were observed (Figure 6D,E). It was apparent by gross examination that the tumor sizes of the DJ4-treated mice were considerably reduced relative to the control mice among the subcutaneously injected OCI-AML3 mouse model (Figure 6E). The mice subcutaneously injected with MV4-11–Luc2–EGFP followed by intraperitoneal DJ4 treatment also had tumor volumes and weights reduced ~3-fold and ~2-fold, respectively, after intraperitoneal DJ4 treatment (*n* = 3) for 3 weeks compared to the control group (*n* = 3) (Figure S7). At the end of the study, the average tumor weight and tumor volume of the control group were approximately 0.55 g and 1200 mm^3^ relative to 0.25 g and 400 mm^3^ of the DJ4-treated group (Figure S7). Additionally, the OCI-AML3–YFP–Luc CDX was administered intravenously, and the effect of DJ4 intraperitoneal treatment was assessed (Figure 6F). There was a moderate benefit reducing the average bioluminescent signal (Figure 6G,H) and increasing the overall survival (Figure 6I) in the DJ4-treated group versus the control group. DJ4-treated mice exhibited a twofold decrease in the bioluminescent signal on day 36 (Figure 6H) and lived 5 days longer than the control group (Figure 6I).

### 3.6. DJ4-Pretreated AML CDX Models Lead to Reduced Leukemia Burden and Prolonged Survival in Mice

**Figure 7:**
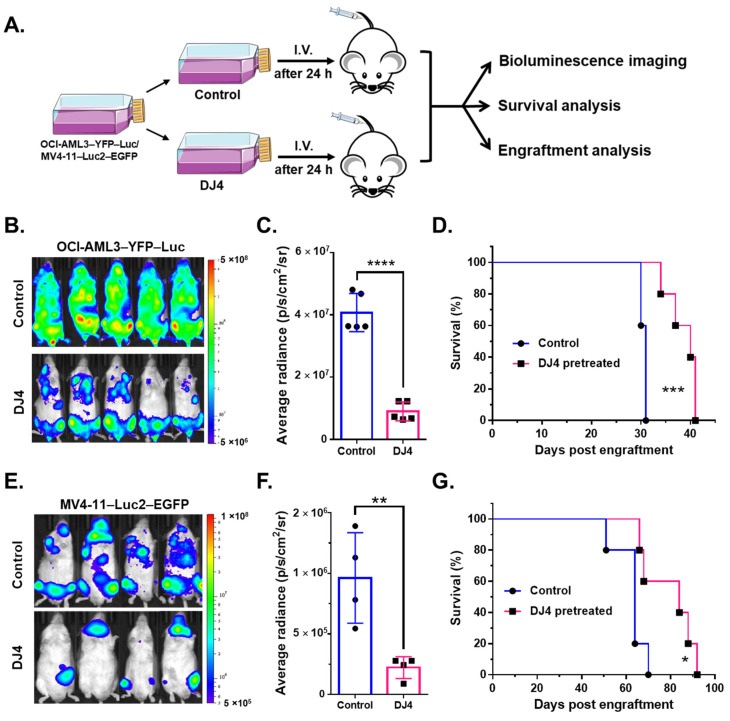
Disease progression and survival analysis of the mice that were intravenously injected with the AML cell lines that were pretreated with DJ4 for 24 h. (**A**) The modified AML cell lines, OCI-AML3–YFP–Luc and MV4-11–Luc2–EGFP, were treated with their respective IC_50_ dose of DJ4 (Figure 1B,C) or with the vehicle for 24 h. The treated cells were then intravenously administered to the NRG-S mice, and the survival advantage was monitored over time without further treatment. The mice were imaged every few days to visualize and quantify disease progression. Flow cytometry analysis on the bone marrow and spleen tissues of the vehicle- and DJ4-treated mice was also performed to track disease progression by measuring the percentage of engraftment of the human cancer cells (see Figure S8). (**B**) Imaging on day 25 of the DJ4-pretreated OCI-AML3–YFP–Luc-injected mice versus the control group. (**C**) Bioluminescence imaging measured by the average radiance (p/s/cm^2^/sr) on day 25 of the DJ4-pretreated OCI-AML3–YFP–Luc-injected group versus the control mice. (**D**) Survival observed among the mice that received the OCI-AML3 cells that were pretreated with DJ4 versus DMSO. (**E**) Imaging on day 38 of the MV4-11–Luc2–EGFP-injected mice that were pretreated with DJ4 versus DMSO. (**F**) The average radiance observed on day 38 in the MV4-11 cells that were pretreated with DJ4 as compared to the DMSO-pretreated mice. (**G**). Kaplan–Meier survival analysis of the DJ4-pretreated MV4-11-injected mice relative to the DMSO-pretreated MV4-11-injected mice. The data are the means ± SD. The values in (**C**,**F**) were assessed by means of the unpaired *t*-test, with ** *p* < 0.01 and **** *p* < 0.0001 considered statistically significant. The results in (**D**,**G**) were examined by means of the Gehan–Breslow–Wilcoxon test, wherein * *p* < 0.05 and *** *p* < 0.005 were considered statistically significant.

The luciferase-expressing modified cell lines were also pretreated with DJ4 in vitro for 24 h and subsequently injected intravenously into the NRG-S mice and monitored without further treatment (Figure 7A). The bioluminescent signal was significantly reduced in the mice that received the DJ4-treated cells (*n* = 4–5) versus those that received the DMSO vehicle-treated cells (*n* = 4–5) (Figure 7B,C,E,F), and the overall survival was greater by 10 to 20 days in the drug treatment group (Figure 7D,G). The mice injected with the DJ4-treated OCI-AML3–YFP–Luc and MV4-11–Luc2–EGFP cells experienced an ~3-fold decrease on day 25 and a ~4-fold reduction on day 38 in bioluminescent intensity, respectively (Figure 7B,C,E,F). Bone marrow and spleen tissues isolated from the control and treatment groups of the disseminated murine studies were analyzed by means of flow cytometry (Figure S8). Flow cytometry analysis indicated a significant reduction in the percentage of hCD45-positive cells in the spleen of the DJ4-pretreated MV4-11 mice by ~1.2-fold relative to the control group (Figure S8A) and a decrease in the hCD45 population by ~2.5-fold versus the control group in the bone marrow of the DJ4-pretreated OCI-AML3-injected mice (Figure S8B).

## 4. Discussion

Patients diagnosed with acute myeloid leukemia exhibit a multitude of genetically diverse molecular alternations or abnormalities [55]. This leads to many different subsets of this disease and complicates treating patients in a generic way. Simplifying the vast molecular landscape of AML to find a suitable therapeutic target requires extensive epigenetic and in vivo investigations. The recent studies have aimed to identify recurrent mutations and establish trends in treatment response among patient samples with targeted inhibitors. This can aid in drug development, determining relationships across multiple signaling pathways, and identifying coinciding abnormalities. Mutation-specific targeted therapeutics such as midostaurin, enasidenib, or ivosidenib toward common AML mutations such as FLT3, IDH1, or IDH2 may help improve current therapy for subgroups manifesting these mutations [56,57,58]. For many older patients, the recent addition of venetoclax, a BLC2-targeting agent, shows improving outcomes. These improvements are, however, transient for most patients, as the majority currently relapse and succumb to the resistant disease.

Expression and mutation profiling studies suggest cancer therapy designed to modulate ROCK activity may improve the prognosis of AML patients. Upregulation of mRNA expression of ROCK1 was observed in thirty-nine AML cell lines from the Cancer Cell Line Encyclopedia (CCLE) database (Figure S1). The Cancer Genome Atlas (TCGA) database of the NIH also indicated ROCK1 overexpression affects the survival of AML patients and correlates well with poor prognosis and survival in AML patients (Figure S2). Loss-of-function RNA interference (RNAi) experiments in AML patient samples identified the knockdown of ROCK1 to reduce the growth and viability of leukemic progenitor cells [59]. Additionally, overexpression of ROCK has been shown to regulate migration and invasion in various cancers [28,60,61]. Potential targeting of the ROCK pathway could modulate cellular proliferation, cell shape and motility, tumor progression and metastasis for therapeutic benefit [41,61]. Mali et al. showed that mutated tyrosine kinase receptors of cells expressing oncogenic forms of KIT, FLT3, and Bcr-Abl constitutively activate the serine/threonine kinase, ROCK [44,62]. Accordingly, AML cell lines (MV4-11 and MOLM-13) and primary AML patient cells which carry the FLT3-ITD or KIT mutation exhibited sensitivity to ROCK inhibitor DJ4 (Figure 1 and Figure 2). Inhibition of the downstream effector of Rho GTPases, ROCK1, by fasudil, H-1152 (dimethylfasudil), or Y27632 resulted in antiproliferative effects on cancer cells suggesting its promise as an AML treatment [44,62,63]. ROCK inhibitors such as fasudil have been established to be safe for administration in humans, but for treatment of other diseases such as cerebral vasospasm, thereby highlighting their clinical tolerability [61,64]. Moreover, the role of the Rho-ROCK pathway as a therapeutic target has been established for various vascular disorders [61,65]. ROCK inhibition may promote normalized tumor vasculature allowing for greater efficacy of chemotherapeutics [61,65], suggesting its potential to work synergistically with the current AML therapeutics. Multiple ROCK inhibitors are needed such as fasudil and presently DJ4 because they may be suitable for certain subsets of AML or more effective in combination with different cancer therapy regimens. Combined inhibition of ROCK and MRCK was previously shown to be more potent in inhibiting actomyosin-regulated functions [41,48,49]. DJ4′s activity to target multiple kinases with similar functions relating to cancer cell migration/invasion [50] builds on the literature relating to the development and design of novel ROCK inhibitors and may even work synergistically with other ROCK inhibitors resulting in a more potent treatment. Future studies will examine DJ4 in combination with other ROCK inhibitors or standard-of-care AML treatments to provide further support for their clinical use in cancer.

Activation of ROCK by Rho results in the phosphorylation of MYPT1, reduced myosin phosphatase activity, and enhanced phosphorylation of the regulatory myosin light-chain 2 substrate [61,66]. Mali et al. demonstrated that PI3K and Rho GTPase regulated activation of the Rho-ROCK pathway results in various leukemic transformations. Stimulation of ROCK1 phosphorylates downstream MLC2 on Ser119, corresponding to actin and myosin changes that promote the acceleration of leukemia cell proliferation; this has been suggested to have consequences for myeloproliferative neoplasms (MPNs) and AML [44,62]. Further, investigations inhibiting ROCK resulted in the diminished proliferation of leukemic cells with activated KIT signaling [44,62,63]. In line with the available literature, immunoblotting analysis of downstream targets of ROCK, MYPT1 and MLC2, upon treating with DJ4 resulted in reduced levels of the phosphorylated substrates as observed in Figure 4. This provides further support of Kale et al.’s observations [50] that DJ4 effectively inhibits ROCK functionality and that the in vivo efficacy observed in this report is in part due to the inhibition of the ROCK/MYPT1/MLC2 axis. The ROCK/MYPT1/MLC2 pathway aids in the regulation of stress fiber assembly, cell adhesion, and motility [61,66]. Inhibition of this pathway has shown suppressed cellular proliferation, invasion, and angiogenesis via in vitro analysis and diminished tumor growth and metastasis formation through in vivo studies, which has been demonstrated in previous works [44,59,61,67,68,69,70,71,72,73].

DJ4 induced potent cytotoxic effects in the AML cell lines with various mutations. DJ4 treatment was also active versus various AML patient-derived cells harboring mutations such as U2AF1, FLT3-ITD, HOXA9/NUP98, NPM1, KIT, and CBL (Figure 1 and Figure 2, Table 1 and Table S1). The cell lines and AML patient cells carry mutations in different oncogenes and signaling pathway genes (Figure 1C and Table 1). AML patient samples are highly heterogenous, with an abundance of mutations, and thus the AML cell lines tended to be more sensitive to DJ4 treatment than the AML patient samples. The observed potency of DJ4 towards various AML cell lines and primary samples with a diverse set of mutations suggests that this is a promising candidate to be incorporated into the standard AML regimen to help many different subsets of AML patients.

In aggressive cases of this disease, the timing to determine the appropriate targeted treatment regimen for a patient can be a limiting factor, therefore finding a targeted therapy that can impart some efficacy and slow disease progression to a wide range of patient groups can be advantageous. The observed diminished leukemic cell proliferation was in part due to the proapoptotic effect induced by DJ4 (Figure 3 and Figures S3 and S4). Therapeutics that induce apoptosis have been shown to be promising candidates toward overcoming chemoresistance and reducing disease progression and work well with appropriate combinations of the standard-of-care drugs [55,74,75]. Interestingly, DJ4 was less active in inhibiting colony formation among normal hematopoietic cells and exhibited only modest hematopoietic toxicity in mice. Therefore, a favorable therapeutic index is postulated, with much greater inhibition of many leukemias than of normal myelopoiesis.

The moderately DJ4-sensitive AML cell line OCI-AML3 and the more sensitive AML cell line MV4-11 (as demonstrated by in vitro assays) were selected to examine the efficacy of DJ4 in murine studies. OCI-AML3 [59] and MV4-11 [44] are also common AML cell lines utilized to examine the efficacy of ROCK inhibitors and, thus, to be consistent with the literature, they were utilized in this report. The mice that were intravenously or subcutaneously injected with OCI-AML3–YFP–Luc or MV4-11–Luc2–EGFP were intraperitoneally treated with DJ4 for three weeks (Figure 6A,F). The reduction of the bioluminescent signal or decreased size of the tumor in volume and weight observed with the cell-derived xenograft models indicated that DJ4 effectively slowed the progression of AML (Figure 6 and Figure S7). This was further demonstrated by the fact that DJ4 treatment resulted in greater overall survival. Efficacy observed after administration of DJ4 to both the disseminated and subcutaneous models suggests the promise of this drug towards different tumor microenvironments. The mice that were intravenously administered DJ4-pretreated AML cells also exhibited a decreased bioluminescent signal, increased survival of 10 and 20 days, and a reduction in the percentage of human CD45 cells in the bone marrow or spleen, indicating a diminished tumor burden (Figure 7 and Figure S8). Treatment of AML mouse models with DJ4 resulted in a significant inhibition of leukemia growth (Figure 6 and Figure 7) without systemic toxicity (Figure 5 and Tables S2–S4). It was unexpected that DJ4 would result in the observed in vivo efficacy due to its poor pharmacokinetics; however, this may be due to the prolonged presence of DJ4 in various tissues such as the liver and tumors. The development of better formulations or variants of DJ4 to improve the pharmacokinetic profile of this treatment may enhance efficacy. Future studies will be focused on gaining insights into the impact of DJ4 on the molecular level to understand in its entirety which signaling pathways or proteins are affected.

ROCK has been shown to regulate proliferation of ITD-FLT3 hematopoietic cells [44,76]. ITD-FLT3 mutations in human AML stem cells are present in 20–30% of AML patients and have been implicated in the poor prognosis and refractory phenotype of this disease [77,78,79,80,81,82]. Onish et al. reported that ITD-FLT3 mutations enhance the leukemic cell migration toward the chemokine Cxcl12 by inhibiting the downregulation of ROCK1 and dephosphorylation of MYPT1 [76,83]. This can result in the hematopoietic stem cells being retained in the bone marrow and protected from AML therapy [76,84,85]. It may be suggested that the improved condition of the MV4-11 mice with DJ4 (Figure 7E,G and Figures S7 and S8) is a result of the decreased ROCK functionality which may be regulating the ITD-FLT3 activity to reduce Cxcl12-induced leukemic cell migration. The decreased AML chemotaxis to this therapy-protective bone marrow microenvironment can allow for the release of leukemia cells and lead to the considerable therapeutic efficacy observed with DJ4 in murine studies. Similarly, disruption of the interaction between the leukemic cells and Cxcl12 by treating FLT3-mutated leukemic cells with the Cxcr4 inhibitor, AMD3465, resulted in the increased proapoptotic activity of an FLT3 inhibitor [76,86]. The scope of this report was to establish the in vivo efficacy of DJ4 and demonstrate the potential of targeting the ROCK/MYPT1/MLC2 pathway in AML. Future investigations will be held to examine signaling networks that may interact with the ROCK signaling pathway in the presence of DJ4. These studies will assess which subsets of AML are most appropriate to incorporate DJ4 into their treatment and can help identify coexisting mutations that DJ4 is also potent towards.

## 5. Conclusions

**Figure 8:**
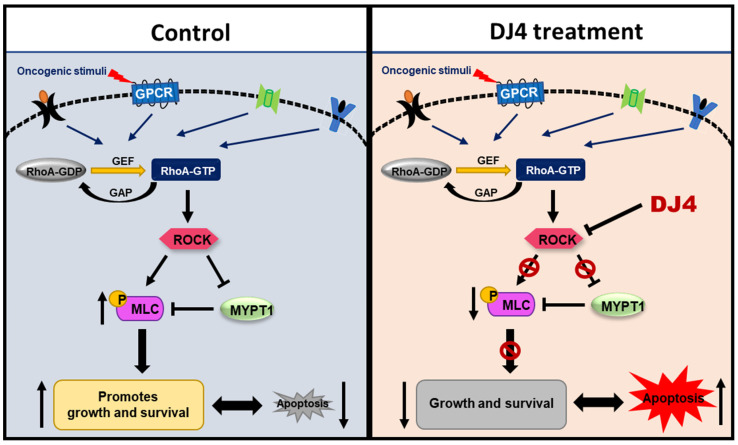
ROCK inhibitors such as DJ4 may reduce the phosphorylation of downstream targets such as MLC2 and MYPT1. Blocking ROCK-related pathways can potentially have therapeutic benefits by restoring proliferation and apoptotic cascades that may be improperly signaling due to malignant transformations arising from cancer.

In this report, the in vivo efficacy of the selective ATP inhibitor of ROCK and MRCK, DJ4, was assessed towards AML. DJ4 was observed to downregulate ROCK functionality via the ROCK/MYPT1/MLC2 pathway (Figure 8) and result in cell death of AML cells, that is in part attributed to inducing apoptosis. AML cell lines and primary AML patient cells with various mutations were considerably sensitive to DJ4 treatment, suggesting its promise to help patients with different subsets of AML. Preclinical therapeutic murine studies showed DJ4 administration reduced leukemia progression and prolonged survival in subcutaneous or disseminated AML mouse models, without limited systemic toxicity. Future experiments will be conducted to establish trends in the treatment response to DJ4 among a larger set of AML patient cases with different cytogenetic backgrounds. This will aid in the determination of which subsets of AML would benefit most from DJ4 treatment. Efforts will be made to continue investigating the effect of DJ4 on ROCK-centered signals and understand its influence on other signaling pathways that may also be contributing to its efficacy. Overall, this study demonstrated the potential of ROCK-targeted therapy to treat patients diagnosed with AML. It also highlighted the need to develop less toxic and more effective chemotherapeutics to overcome the poor prognosis and chemoresistance that is frequently associated with AML patients.

## Figures and Tables

**Table 1:** AML patient information and the corresponding IC_50_ (µM) values for DJ4.

Code	Age	Gender	WBC (×10,000/µL)	Cytogenetics	Molecular Data	DJ4 IC_50_ (µM)
990	69	M	106.66	46,XY,del(13)(q12q14)[2]/46,XY[18]	U2AF1	0.264
1265	74	M	217.36	46,XY,t(7;11)(p15;p15)	FLT3-ITD, HOXA9/NUP98 FUSION	0.504
1241	50	F	180.40	46,XX	NPM1, FLT3-ITD	2.769
1172	46	F	149.23	46,XX	FLT3-ITD	5.055
1044	70	M	36.4	46,XY,i(17)(q10)[12]/47,idem,+13[2]/47,XY, +mar[6]	CBL, APC, SALL4, ASXL1, SETBP1, SRSF2, FLT3-ITD, ESCO2	5.142
1103	41	F	247.25	46,XX	FLT3-ITD, NOTCH1, PTPN11	5.167
1290	74	M	99.07	46,XY	ASXL1, DNMT3A, IDH1, KRAS, NRAS, RUNX1	5.616
1341	83	M	201.00	46,XY,t(1;3)(p34.1;q27), t(2;18)(q31;q11.2), del(11)(p11.2p15)	CBL, KIT	5.765
1099	86	M	141.02	46,XY	N/A	13.430

## Data Availability

The datasets analyzed during the current study are available from the corresponding author upon request or using the link provided in the Supplementary Materials.

## Supplementary Materials

The following are available online at https://www.mdpi.com/article/10.3390/cancers13194889/s1, Figure S1: ROCK 1 kinase A. and ROCK 2 kinase B. were found to be highly expressed in different cancer cell lines including among 39 AML cell lines from CCLE database, Figure S2: Survival plots of AML patients with low and high expression of ROCK 1 kinase and ROCK 2 kinase accessed from the BloodSpot database, Figure S3: Cell cycle analysis was performed on human AML cell lines in the presence of increasing concentrations of DJ4 for 24 h, Figure S4: The percent of apoptosis was measured as the percent of Annexin V-positive cells upon treating AML primary samples with DJ4, Figure S5. The percent of YFP or GFP expression quantified in labeled cell lines versus their respective unlabeled control cells by flow cytometry, Figure S6: Cell proliferation quantified by MTS assay upon treating labeled cell lines and their respective unlabeled cell lines with DJ4, Figure S7: Efficacy was imparted by intraperitoneal DJ4 administration (10 mg/kg) for three weeks on the subcutaneously injected MV4-11–Luc2–EGFP model, Figure S8: Flow cytometry analysis was performed on the bone marrow and spleen tissues harvested from the modified cell lines that were pretreated with DJ4 or DMSO, Figure S9: All the whole western blot figures, Table S1: Various human AML cell lines were treated with DJ4 and the IC50 (µM) values were determined by the MTS assay, Table S2: Mice were interperitoneally administered DMSO (*n* = 5) or 10 mg/kg DJ4 (*n* = 5) for 2.5 weeks and the weights of the mice were recorded over time, Table S3: Complete blood count (CBC) values, and Table S4: Clinical Chemistry values.
