# Supplementary material for: DJ4 Targets the Rho-Associated Protein Kinase Pathway and Attenuates Disease Progression in Preclinical Murine Models of Acute Myeloid Leukemia
Source: Cancers (Basel). 2021 Sep 29;13(19):4889. doi: 10.3390/cancers13194889 (PMC8508452; doi:10.3390/cancers13194889)
Supplement: Supplementary file 1 [file cancers-13-04889-s001.zip › Supplementary Material SF1-SF8, ST1-ST4.pdf]

Supplementary Materials

DJ4 Targets the Rho-associated Protein Kinase Pathway and Attenuates Disease Progression in Preclinical Murine Models of Acute Myeloid Leukemia

Upendarrao Golla [1,2,†], Melanie A. Ehudin [3,†], Charyguly Annageldiyev [1,2], Zheng Zeng [4], Diwakar Bastihalli Tukaramrao [3], Anna Tarren [1,2], Abhijit A. Date [5], Irina Elcheva [3], Arthur Berg [2], Shantu Amin [2,4], Thomas P. Loughran, Jr. [6,7], Mark Kester [4,7], Dhimant Desai [2,4], Sinisa Dovat [3,*], David Claxton [1,2] and Arati Sharma [1,2,4,*]

[1] Department of Medicine, Division of Hematology and Oncology, Pennsylvania State University College of Medicine, Hershey, PA 17033, USA; ugolla@pennstatehealth.psu.edu (U.G.); cannageldiyev@pennstatehealth.psu.edu (C.A.); atarren@pennstatehealth.psu.edu (A.T.); dclaxton@pennstatehealth.psu.edu (D.C.)

[2] Penn State Cancer Institute, Pennsylvania State University College of Medicine, Hershey, PA 17033, USA; asb17@psu.edu (A.B.); samin@pennstatehealth.psu.edu (S.A.); ddesai@pennstatehealth.psu.edu (D.D.)

[3] Division of Hematology and Oncology, Department of Pediatrics, Pennsylvania State University College of Medicine, Hershey, PA 17033, USA; mehudin@pennstatehealth.psu.edu (M.A.E.); dbastihallitukaramra@pennstatehealth.psu.edu (D.B.T.); ielcheva@pennstatehealth.psu.edu (I.E.)

[4] Department of Pharmacology, Pennsylvania State University College of Medicine, Hershey, PA 17033, USA; beigangmm@hotmail.com (Z.Z.); mk5vq@virginia.edu (M.K.)

[5] The Daniel K. Inouye College of Pharmacy, University of Hawaii, Hilo, HI 96720, USA; dateabhi@hawaii.edu

[6] Department of Medicine, Division of Hematology and Oncology, University of Virginia School of Medicine, Charlottesville, VA 22903, USA; tl7cs@virginia.edu

[7] Department of Medicine, Division of Hematology and Oncology, University of Virginia Cancer Center, Charlottesville, VA 22903, USA

* Correspondence: sdovat@pennstatehealth.psu.edu (S.D.); asharma@pennstatehealth.psu.edu (A.S.)

† These authors contributed equally to this work.

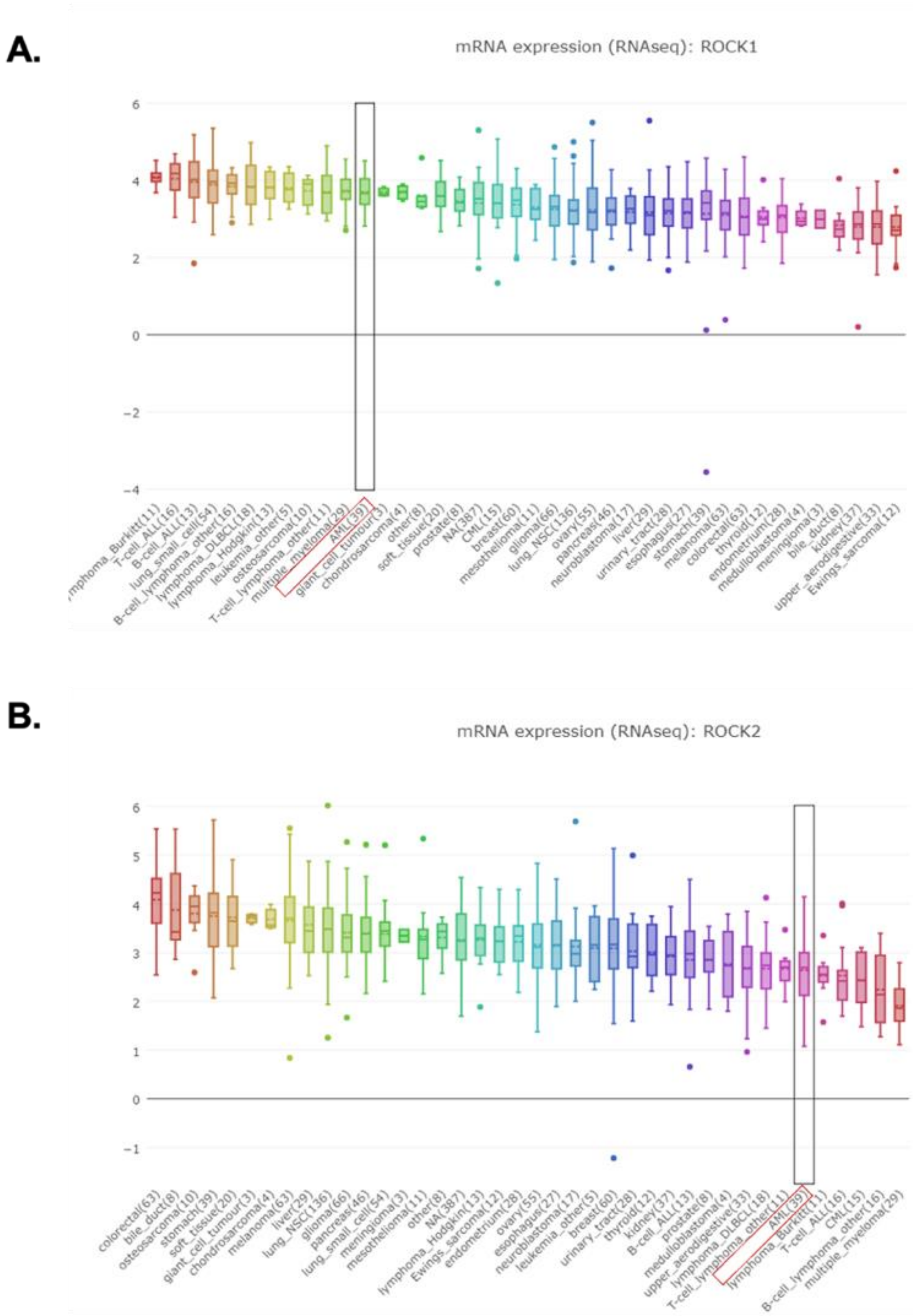

Figure S1. ROCK 1 kinase **A**. and ROCK 2 kinase **B**. were found to be highly expressed in different cancer cell lines including among 39 AML cell lines (indicated with red box) from Broad Institute Cancer Cell Line Encyclopedia (CCLE) database [1].

A.

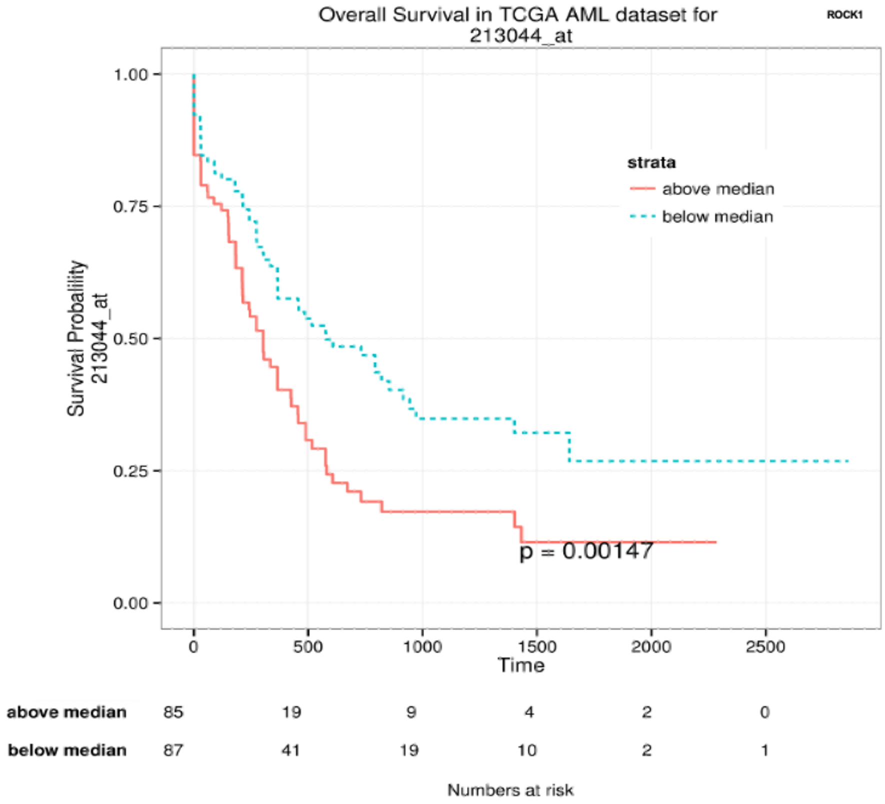

B.

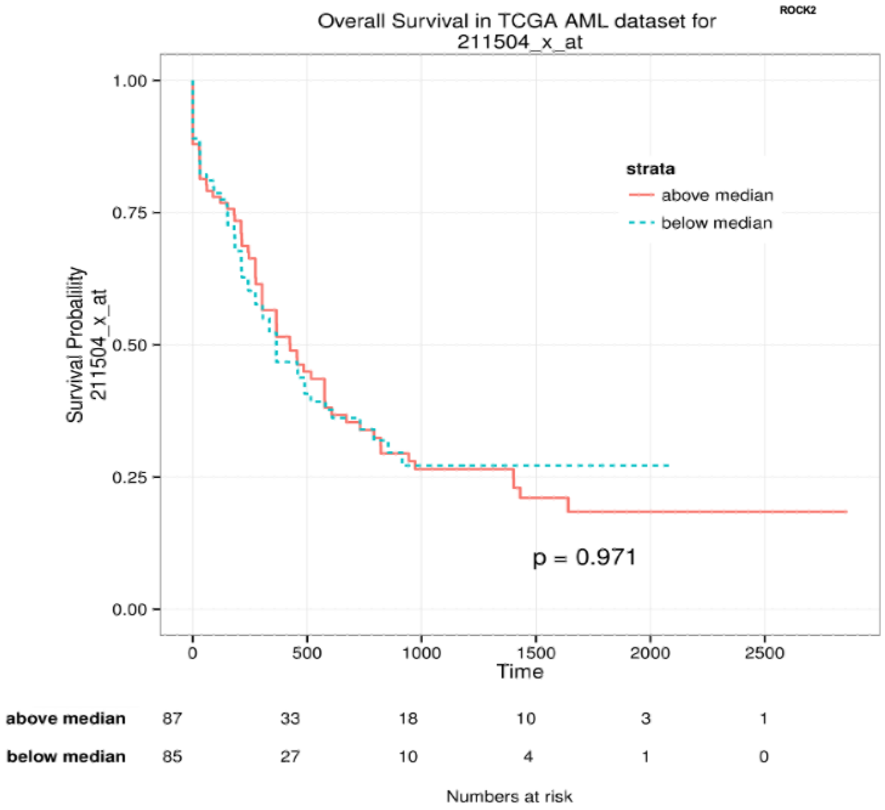

Figure S2. Survival plots of AML patients with low (in blue) and high expression (in red) of ROCK 1 kinase **A.** and ROCK 2 kinase **B.** accessed from the BloodSpot database (The Cancer Genome Atlas Program (TCGA) AML dataset versus normal) [2].

Table S1. Various human AML cell lines were treated with increasing concentrations of DJ4 for 24 h and the IC_{50} (µM) values were determined by the MTS assay (Figure 1B).

Cell line	IC_{50} ± SD (µM)
MV4-11	0.05 ± 0.02
MOLM-13	0.15 ± 0.03
OCI-AML2	0.63 ± 0.07
OCI-AML3	0.81 ± 0.12
HL-60	0.93 ± 0.08
U937	1.68 ± 0.70

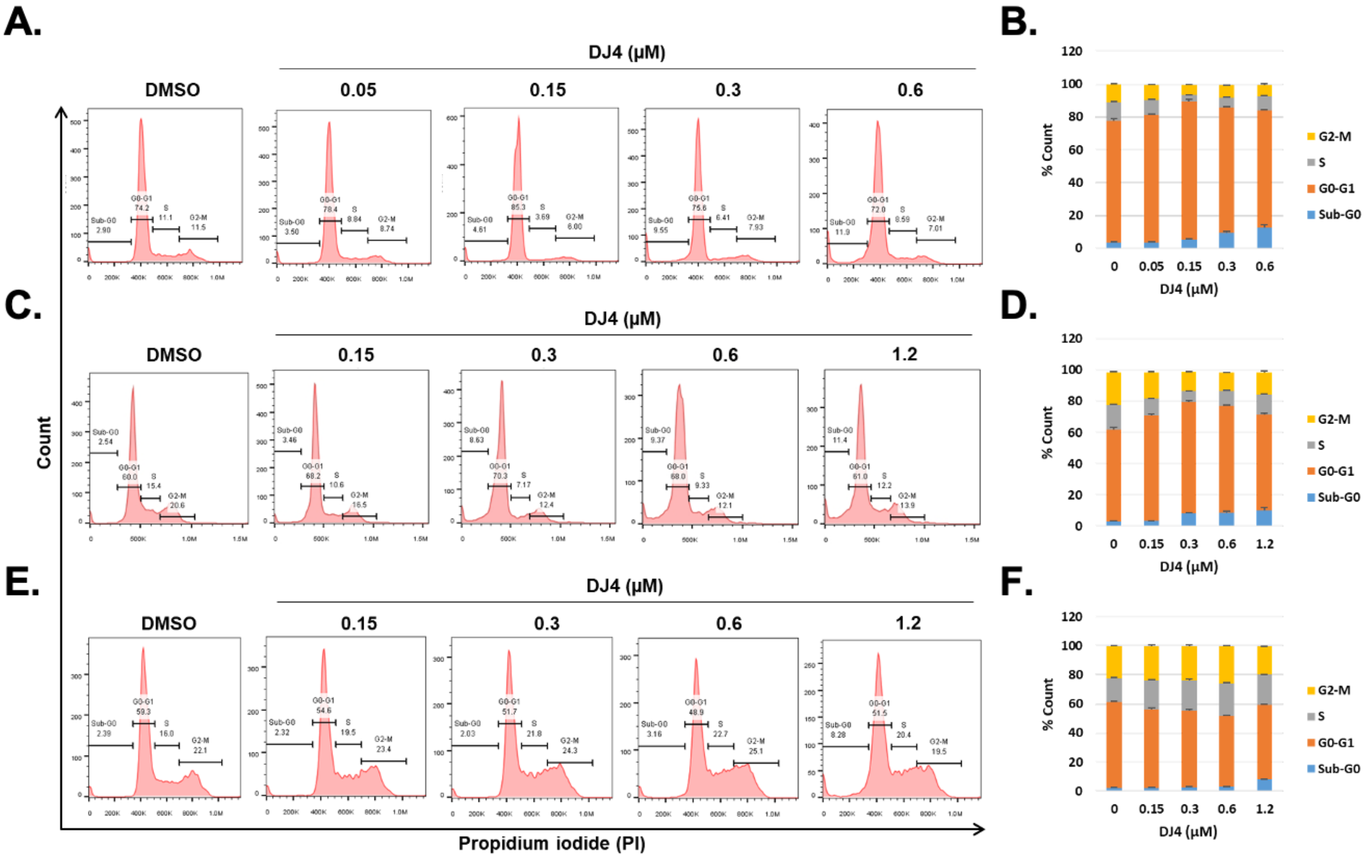

Figure S3. Cell cycle analysis was performed on human AML cell lines in the presence of increasing concentrations of DJ4 for 24 h. **A**. Changes in the cell cycle phases of MV4-11 cells upon DJ4 treatment. **B**. Bar graph representation showing changes in the MV4-11 cell cycle phases with various concentrations of DJ4. **C**. Analysis of the cell cycle phases in the presence of DJ4 treatment on MOLM-13 cells. **D**. Bar graph representation demonstrating changes in the MOLM-13 cell cycle phases upon treating with increasing DJ4 concentrations. **E**. OCI-AML3 cells treated with DMSO or indicated doses of DJ4 for 24 h prior to cell cycle analysis. **F**) Bar graph representation of OCI-AML3 cell cycle phases with varying concentrations of DJ4.

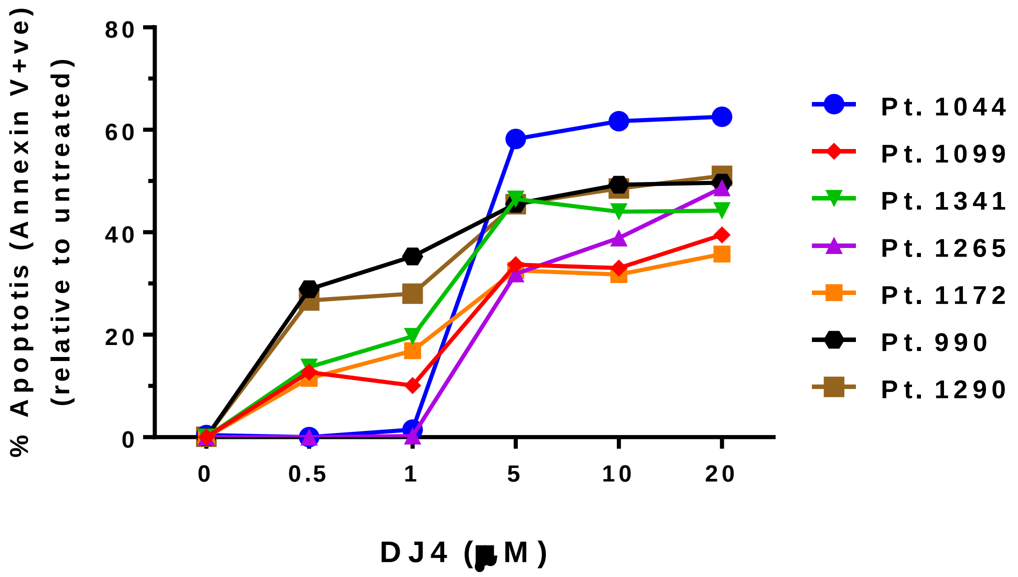

Figure S4. The percent of apoptosis was measured as the percent of Annexin V-positive cells upon treating AML primary samples with increasing concentrations of DJ4. These values were normalized relative to the respective untreated cells to account for the baseline apoptotic population and to aid in the comparison of the various samples.

Table S2. Mice were interperitoneally administered DMSO (n = 5) or 10 mg/kg DJ4 (n = 5) for 2.5 weeks and the weights of the mice were recorded over time. The average body weight (g) for the mice were calculated to monitor the effect of DJ4 versus control mice over time. Data is represented as Mean ± SD.

**Day**	**Average Body Weight (g)**	
	**Control**	**DJ4**
**0**	17.80 ± 0.84	18.20 ± 0.45
**1**	17.40 ± 0.55	17.20 ± 0.84
**2**	17.20 ± 0.84	16.60 ± 0.55
**4**	17.20 ± 0.45	17.00 ± 1.23
**7**	18.52 ± 0.62	18.78 ± 0.65
**8**	18.68 ± 0.92	19.08 ± 0.71
**9**	18.38 ± 0.73	18.28 ± 0.98
**10**	17.80 ± 0.84	18.20 ± 0.45
**11**	17.80 ± 0.45	17.80 ± 0.45
**14**	18.40 ± 1.52	18.60 ± 0.89
**15**	18.40 ± 0.55	17.60 ± 0.89
**16**	18.00 ± 0.00	17.60 ± 0.89

Table S3. Complete blood count (CBC) values.

Complete Blood Count			
	**Control**	**DJ4**	**Normal range**
**WBC ($10^3/mm^3$)**	5.68 ± 1.25	6.84 ± 2.43	8.20 ± 3.90
**RBC ($10^6/mm^3$)**	9.56 ± 0.83	8.28 ± 1.18	9.10 ± 0.80
**Hgb (g/dl)**	14.54 ± 1.17	12.04 ± 1.74	15.50 ± 1.10
**HCT (vol%)**	46.08 ± 3.96	39.22 ± 5.74	44.10 ± 3.00
**MCV (mg/dL)**	48.16 ± 0.34	47.34 ± 1.22	49.00 ± 1.30
**MCH (pg)**	15.22 ± 0.23	14.54 ± 0.46	17.10 ± 0.70
**MCHC (g/dL)**	31.60 ± 0.71	30.76 ± 0.45	35.20 ± 0.70
**Platelets (fl)**	1031.20 ± 320.41	1106.60 ± 309.32	900.00 ± 311

Table S4. Clinical Chemistry values.

	**Control**	**DJ4**
**GLU (mg/dL)**	254.00 ± 31.91	223.60 ± 19.07
**CREA (mg/dL)**	0.10 ± 0.00	0.10 ± 0.00
**BUN (mg/dL)**	18.75 ± 1.71	15.75 ± 1.50
**PHOS (mg/dL)**	11.35 ± 1.64	12.37 ± 2.86
**CA (mg/dL)**	10.50 ± 0.42	11.14 ± 1.94
**TP (g/dL)**	5.33 ± 0.33	6.08 ± 2.55
**ALB (g/dL)**	2.78 ± 0.21	2.94 ± 1.17
**ALT (U/L)**	88.00 ± 53.98	113.20 ± 47.31
**ALKP (U/L)**	116.50 ± 27.64	100.00 ± 26.85
**TBIL (mg/dL)**	0.53 ± 0.05	0.53 ± 0.10
**CHOL (mg/dL)**	90.50 ± 8.35	81.25 ± 13.35
**AMYL (U/L)**	1756.00 ± 124.13	1785.50 ± 142.13

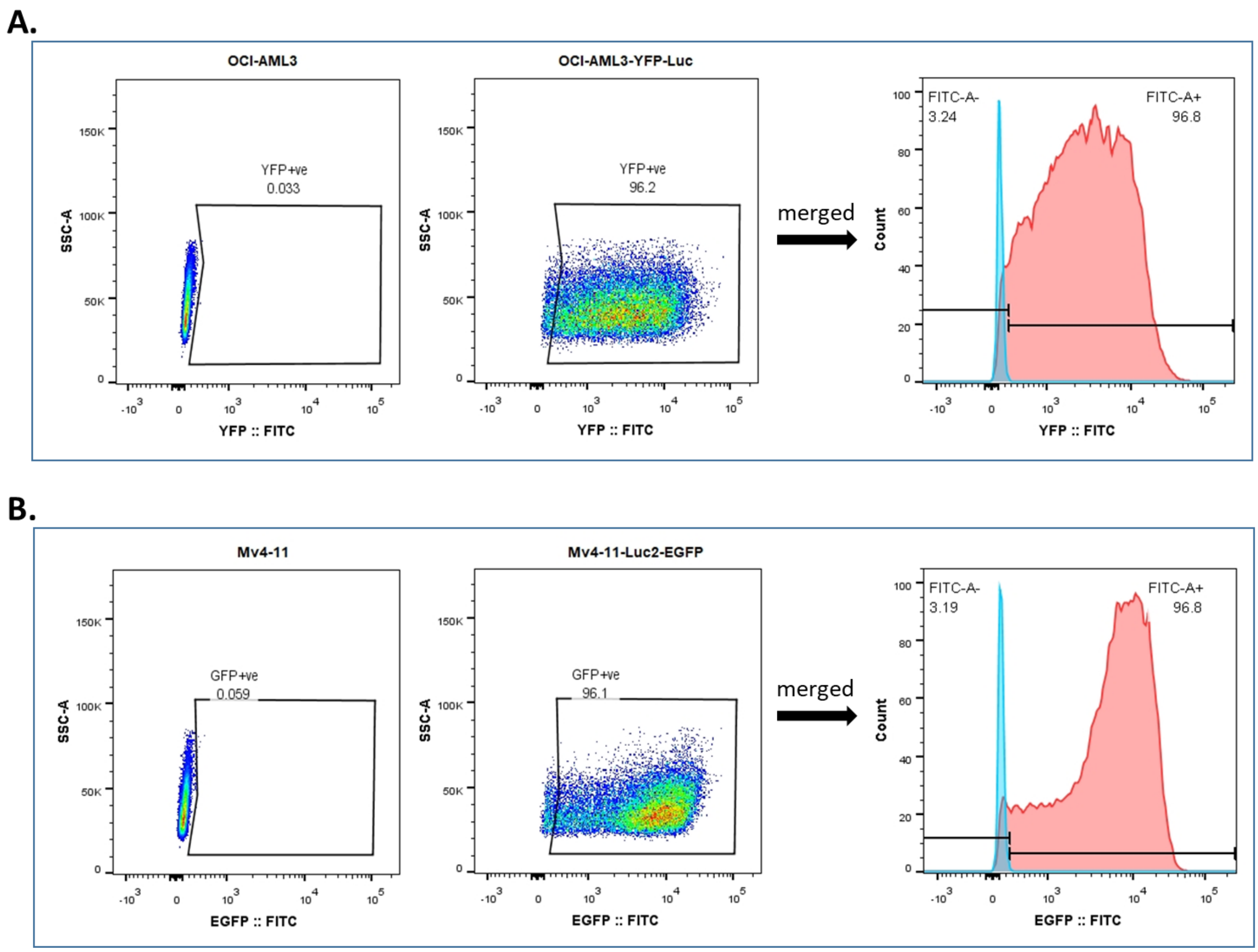

Figure S5. The percent of YFP or GFP expression quantified in OCI-AML3-YFP-Luc (A) and MV4-11-Luc2-EGFP (B) versus their respective unlabeled control cells by flow cytometry.

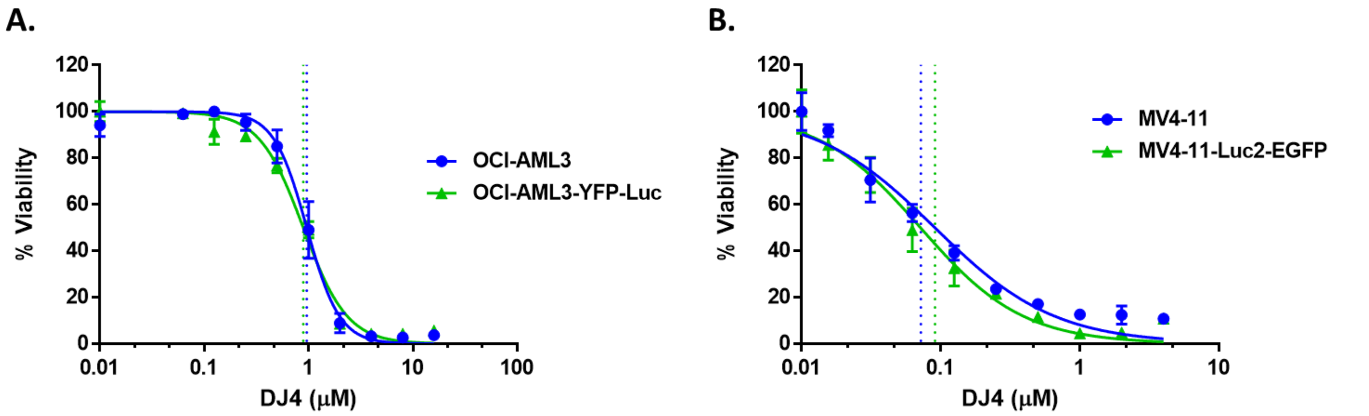

Figure S6. Cell proliferation quantified by MTS assay upon treating OCI-AML3-YFP-Luc (A) and MV4-11-Luc2-EGFP (B) and their respective unlabeled cell lines with increasing concentrations of DJ4 for 24 h. The dotted vertical line indicates the IC_{50} value.

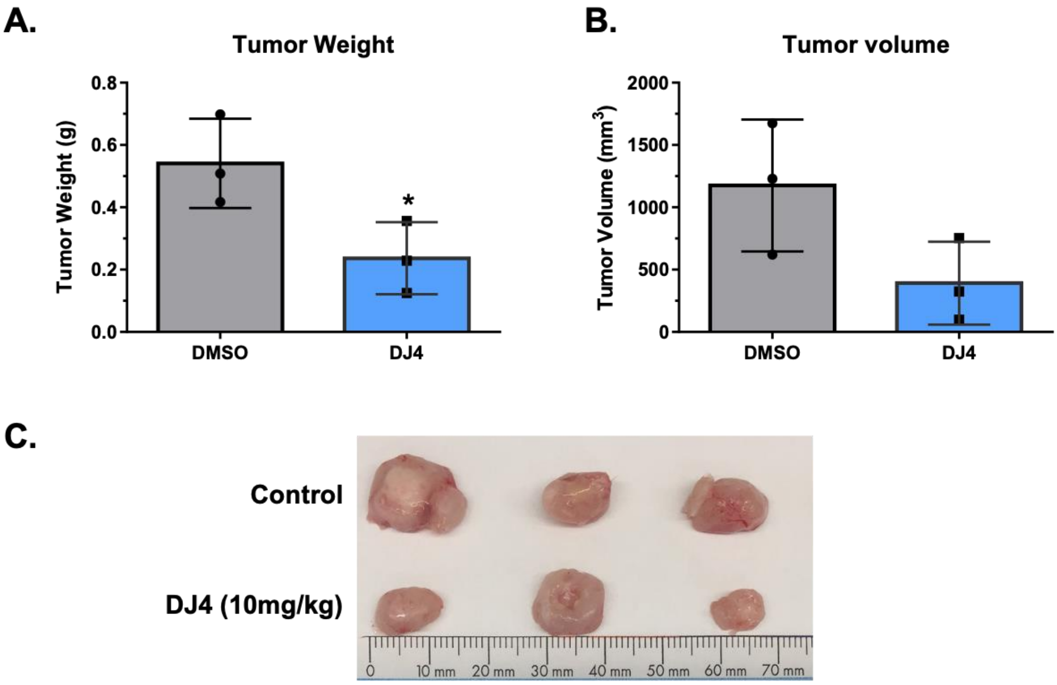

Figure S7. Efficacy was imparted by intraperitoneal DJ4 administration (10 mg/kg) for three weeks on the subcutaneously injected MV4-11-Luc2-EGFP model. Endpoint analysis included harvesting the tumors from the DJ4 and DMSO treated groups and measuring the tumor weight and volume. **A**. Tumor weights (g) were observed among the DJ4 treated mice relative to the control group. **B**. Volume of the tumors (mm^3) to monitor the effect of DJ4 treatment. **C**. Gross examination of the isolated tumors at the end of the study. *$P<0.05$ (unpaired t-test) was considered statistically significant.

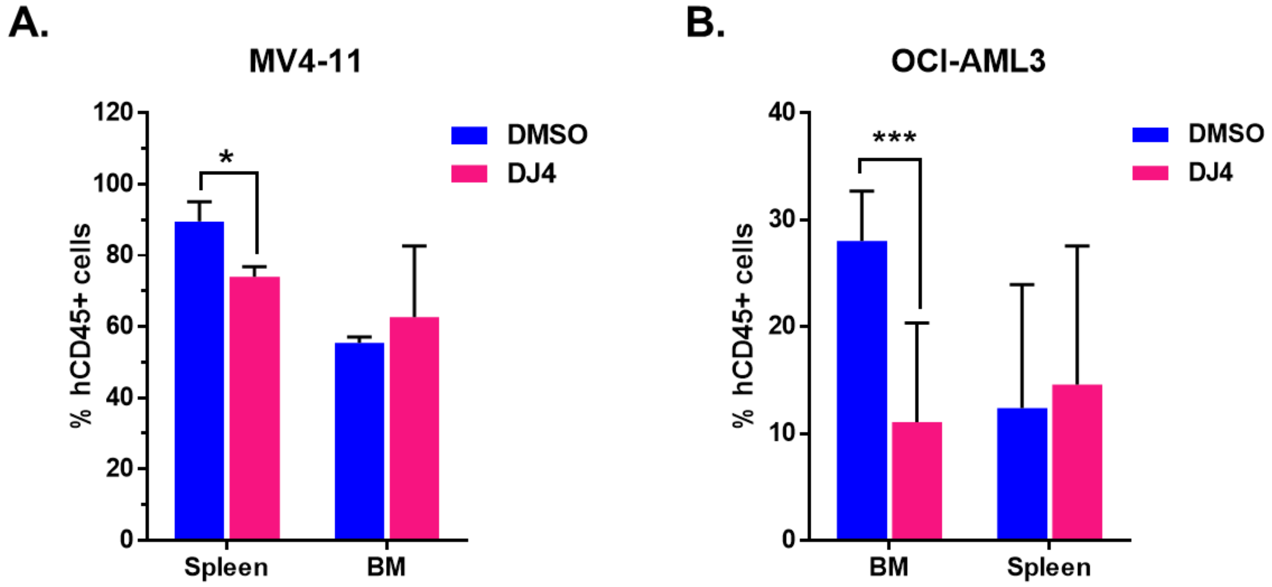

Figure S8. Flow cytometry analysis was performed on the bone marrow and spleen tissues harvested from the modified cell lines, OCI-AML3-YFP-Luc and MV4-11-Luc2-EGFP, that were pretreated with DJ4 or DMSO. The percent of engraftment was determined by quantifying the percent of the human CD45 positive population versus the sum of the mouse and human CD45 positive populations. **A**. The percent of the human CD45 population was determined by analyzing the spleen and bone marrow (BM) tissues from mice engrafted with DMSO and DJ4 pretreated MV4-11 cells. **B**. The percent of positive human CD45 cells was accessed by examining the spleen and bone marrow tissues of DMSO and DJ4 pretreated OCI-AML3 modified cells. Data was assessed by t-test wherein *$P<0.05$ and ***$P<0.005$ considered statistically significant when compared to DMSO control.

References

1. Barretina, J.; Caponigro, G.; Stransky, N.; Venkatesan, K.; Margolin, A.A.; Kim, S.; Wilson, C.J.; Lehár, J.; Kryukov, G.V.; Sonkin, D.; et al. The Cancer Cell Line Encyclopedia enables predictive modelling of anticancer drug sensitivity. *Nature* **2012**, *483*, 603-607, doi:10.1038/nature11003.
2. Bagger, F.O.; Kinalis, S.; Rapin, N. BloodSpot: a database of healthy and malignant haematopoiesis updated with purified and single cell mRNA sequencing profiles. *Nucleic Acids Research* **2018**, *47*, D881-D885, doi:10.1093/nar/gky1076.

© 2021 by the authors. Licensee MDPI, Basel, Switzerland. This article is an open access article distributed under the terms and conditions of the Creative Commons Attribution (CC BY) license (http://creativecommons.org/licenses/by/4.0/).
