# Supplementary material for: DJ4 Targets the Rho-Associated Protein Kinase Pathway and Attenuates Disease Progression in Preclinical Murine Models of Acute Myeloid Leukemia
Source: Cancers (Basel). 2021 Sep 29;13(19):4889. doi: 10.3390/cancers13194889 (PMC8508452; doi:10.3390/cancers13194889)
Supplement: Supplementary file 1 [file cancers-13-04889-s001.zip › Supplementary Material SF9.pptx]

## Slide 1
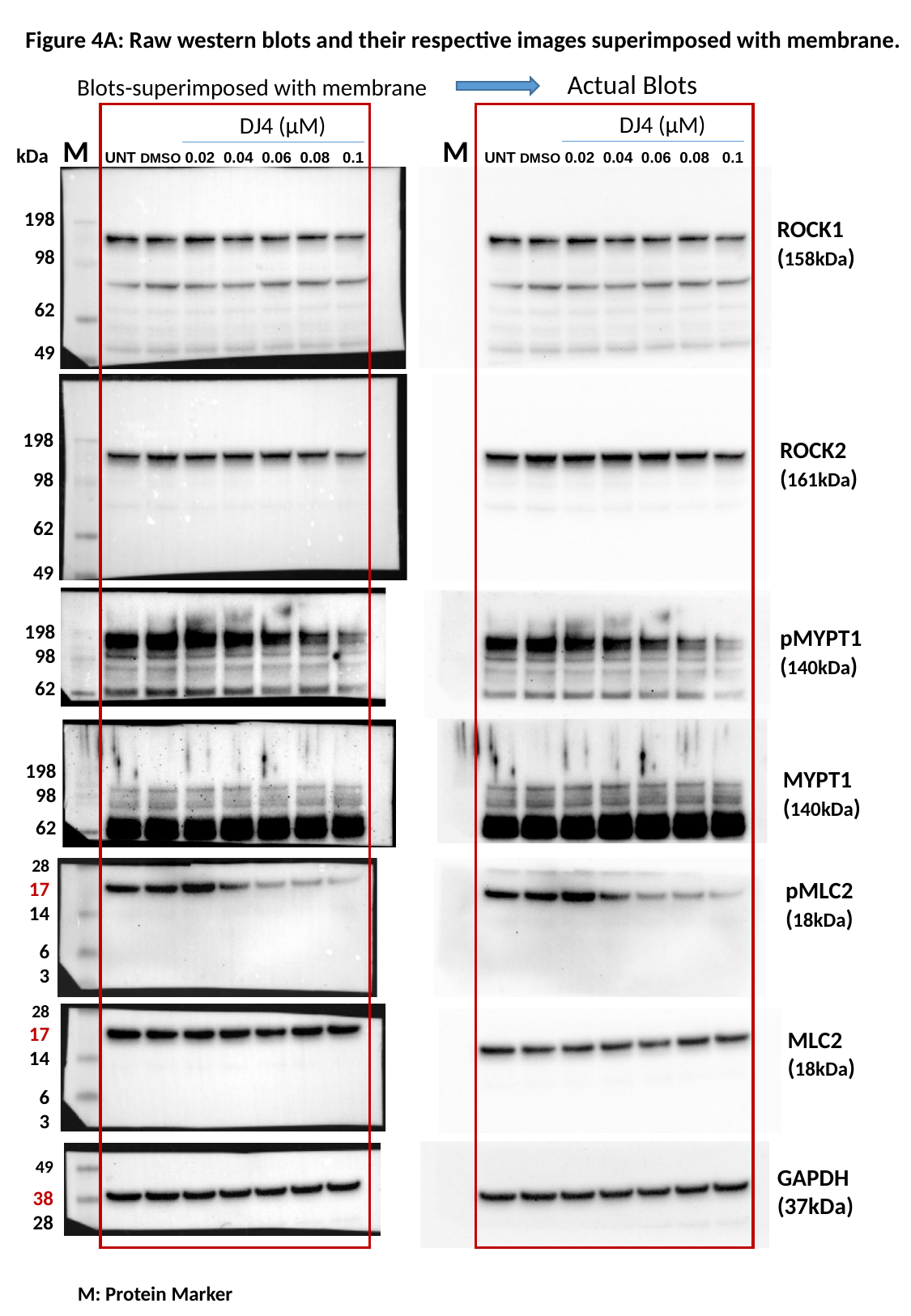

Figure 4A: Raw western blots and their respective images superimposed with membrane.
Actual Blots
Blots-superimposed with membrane
DJ4 (µM)
DJ4 (µM)
M
M
kDa
UNT DMSO 0.02 0.04 0.06 0.08 0.1
UNT DMSO 0.02 0.04 0.06 0.08 0.1
198
98
62
49
ROCK1
(158kDa)
198
98
62
49
ROCK2
(161kDa)
198
98
62
pMYPT1
(140kDa)
198
98
62
MYPT1
(140kDa)
28
17
14
6
3
pMLC2
(18kDa)
28
17
14
6
3
MLC2
(18kDa)
49
38
28
GAPDH
(37kDa)
M: Protein Marker

## Slide 2
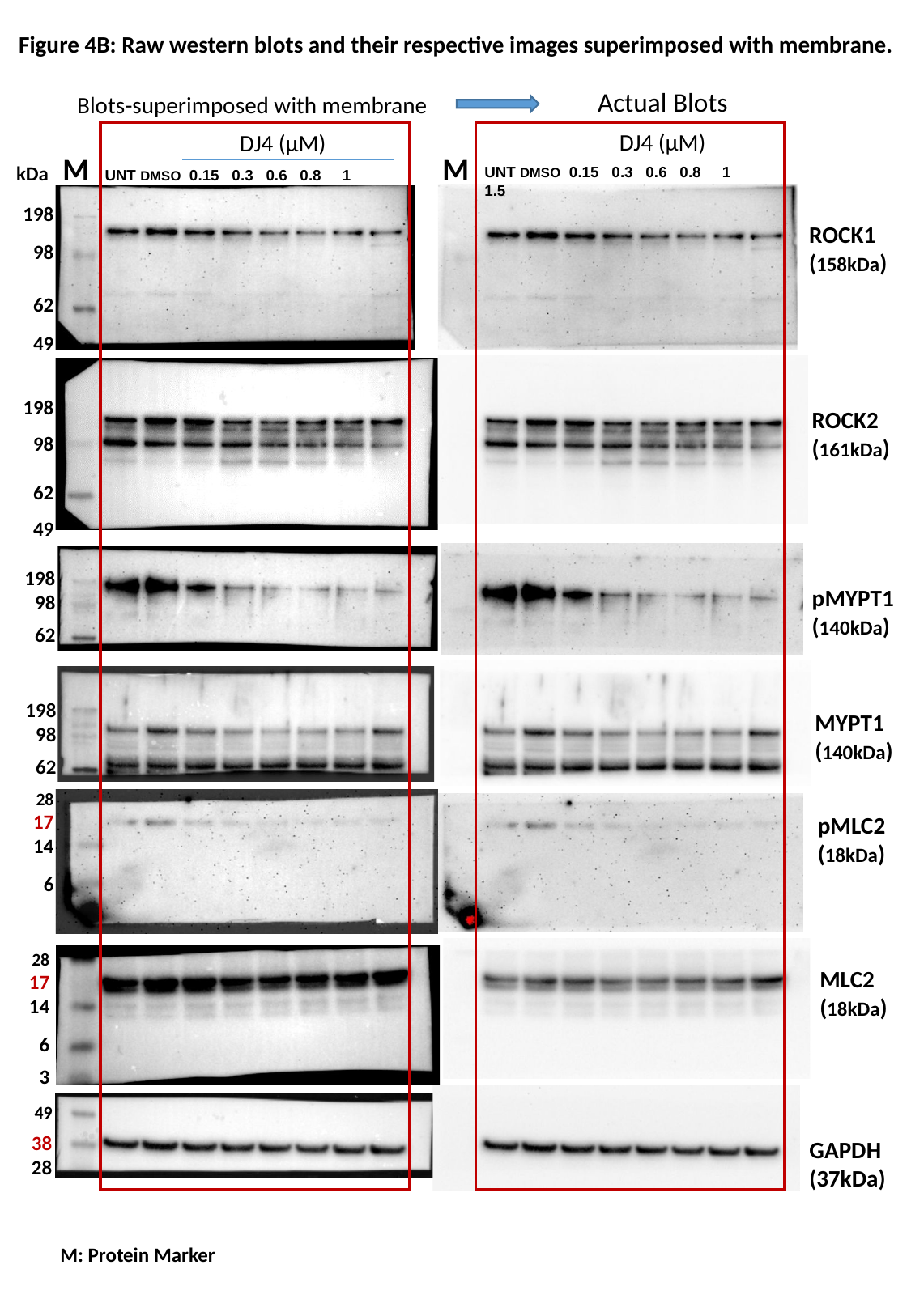

Figure 4B: Raw western blots and their respective images superimposed with membrane.
Actual Blots
Blots-superimposed with membrane
DJ4 (µM)
DJ4 (µM)
M
M
UNT DMSO 0.15 0.3 0.6 0.8 1 1.5
UNT DMSO 0.15 0.3 0.6 0.8 1 1.5
kDa
198
98
62
49
ROCK1
(158kDa)
198
98
62
49
ROCK2
(161kDa)
198
98
62
pMYPT1
(140kDa)
198
98
62
MYPT1
(140kDa)
28
17
14
6
pMLC2
(18kDa)
28
17
14
6
3
MLC2
(18kDa)
49
38
28
GAPDH
(37kDa)
M: Protein Marker
